# Temporal Trends in the Prevalence of Frailty in England From 2002 to 2022, With Forecasts to 2040: Longitudinal Secondary Analysis of a National Cohort Study

**DOI:** 10.2196/98665

**Published:** 2026-11-03

**Authors:** Dongfeng Tang, Katie Jane Sheehan, Simon Conroy, Aicha Goubar, Julie Whitney, Yanzhong Wang, Matthew D L O'Connell

**Affiliations:** 1 School of Life Course and Population Sciences, Faculty of Life Science and Medicine, King's College London, Great Maze Pond, Guy's Campus, London, England, SE1 1UL, United Kingdom, 44 07522762135; 2 Center for Bone & Joint Health, Blizard Institute, Queen Mary University of London, London, England, United Kingdom; 3 Medical Research Council Unit for Lifelong Health and Ageing, University College London, London, England, United Kingdom; 4 Royal London Hospital, London, England, United Kingdom; 5 Wolfson Institute of Population Health, Queen Mary University of London, London, England, United Kingdom; 6 Department of Clinical Gerontology, King's College Hospital NHS Foundation Trust, London, England, United Kingdom; 7 NIHR Applied Research Collaboration (ARC) South London, London, England, United Kingdom

**Keywords:** frailty, aging, prevalence, trend, projection

## Abstract

**Background:**

The increasing life expectancy provides an opportunity for healthy longevity, but frailty is a potential challenge. Existing studies of frailty prevalence in England are largely cross-sectional, use heterogeneous frailty definitions, and rarely break down population-level drivers or project future burden, limiting their use for health and social care planning.

**Objective:**

This study aimed to explore temporal trends of frailty prevalence and project scenario-based future prevalence up to 2040 for the community-dwelling population aged 50 years and older in England.

**Methods:**

Prevalence of frailty (measured using a frailty index) in the overall sample and subgroups of age, sex, educational level, and marital status from 2002 to 2022 was estimated among the community-dwelling population aged 50 years and older using 10 waves of nationally representative data from the English Longitudinal Study of Ageing (ELSA; n=6226 to 11,218 across waves). The number of individuals with frailty was also derived in each year. Joinpoint regression models were used to explore temporal trends of frailty prevalence. A linear probability regression model was performed using pooled data from 10 waves of the ELSA to explore driving factors and project future prevalence. Decomposition analysis was used to estimate the contribution of individual factors to the change in prevalence.

**Results:**

A statistically nonsignificant (*P*=.61) increase of 2.3 percentage points from 15.5% (1738/11,218) in 2002 to 17.8% (1108/6226) in 2022 was observed, with the number of individuals with frailty growing from 2,560,122 to 3,791,038 among community-dwelling adults aged 50 years and older in England. Those with advanced age, who were female, had a lower educational level, and/or were single had a consistently higher prevalence of frailty. Opposing contributions netted to this overall change: improvements in sociodemographic conditions and educational attainment and healthier lifestyle behaviors offset 222.5%, 94.9%, and 51.6% of the observed increase, respectively. Under scenario-based projections, prevalence was estimated at 20.5% (95% CI 19.2%-21.9%) in 2040, corresponding to 5,213,689 people with frailty.

**Conclusions:**

Despite a modest increase in prevalence among the community-dwelling population aged 50 years and older, the substantial growth in the absolute number of individuals with frailty indicates a likely rise in future care demands. The study contributes a 20-year trend, decomposition, and forecast framework for community-dwelling older adults. Findings support prioritizing broader sociodemographic development alongside educational attainment and healthy lifestyles, with potential policy approaches including income and social protection, housing improvements, access to health care, and prevention. Key limitations include self-reported measures, exclusion of care home residents, and potential influence of unmeasured effects.

## Introduction

The increasing life expectancy in high-income countries since at least 1900 represents one of the remarkable achievements of modern society, reflecting significant advances in social and economic development [1,2]. This longevity offers immense potential for societal contribution and enriched later life, and therefore, it is important to promote healthy life expectancy. In 2018, the UK government set a responsive goal to increase healthy life expectancy by at least 5 years by 2035 [3]. However, frailty is a potential challenge to healthy longevity. It is a geriatric syndrome associated with aging, characterized by multiple functional declines and increased vulnerability to stressors and associated with various adverse health outcomes, including disability, hospitalization, admission to nursing homes, and mortality [4,5]. Therefore, it is a critical target for innovative approaches to meet growing health and social care needs to ensure that those extended years are lived in good health. There is a need for health and care services to balance priorities, needs, and resources to support a response to population frailty [6]. Such informed policymaking and planning require a better understanding of the present and potential future prevalence of frailty and associated explanatory factors.

Frailty prevalence in the United Kingdom has been reported to range from 4.3% to 38.9%, largely due to differences in frailty measurement tools and study characteristics across the literature [7-10]. However, most of the studies are cross-sectional, limiting understanding of how this prevalence has changed over time [7-9]. In the study by Walsh et al [10] based on routine primary care data, the prevalence of frailty was found to increase from 26.5% to 38.9% (electronic frailty index [FI]) from 2006 to 2017 among adults aged 50 years and older. While this suggests an upward trend, understanding how this trend may develop into the future requires consideration of the evolving picture of population-level risk factors. Although risk factors for within-individual changes in frailty have been explored [10,11], the driving factors for population-level changes in frailty prevalence have received little attention. Studies describing temporal trends in the prevalence of age-related disorders, including musculoskeletal disorders, Parkinson disease, and age-related macular degeneration, have identified contributors such as demographic shifts, epidemiological transitions, and changes in exposures to risk factors [12-14]. These findings collectively suggest that a multidimensional lens is necessary to understand the factors driving the temporal trends in frailty prevalence. Moreover, an understanding of the evolution of frailty into the future is required to inform policy planning and target interventions.

Understanding how frailty prevalence has changed and will change is central to planning health and social care for an aging population. This study aimed to examine the temporal trends in frailty prevalence among the community-dwelling population aged 50 years and older in England from 2002 to 2022 and project the future prevalence up to 2040. It also sought to identify key factors driving changes in population frailty and estimate their effects on future projections, thereby providing a basis for targeting prevention and allocating future care resources.

## Methods

### Study Design and Participants

Ten waves of data (baseline commenced in 2002-2003, and follow-up surveys were conducted every 2 years up to 2018-2019, with another follow-up in 2021-2023) from the English Longitudinal Study of Ageing (ELSA) were used (n=6226 to 11,218 across waves). The ELSA is a representative survey of the population aged 50 years and older living in private households in England, involving the collection of economic, social, psychological, cognitive, health, biological, and genetic data [15]. Participants in the ELSA were initially recruited from households that participated in the Health Survey for England in 1998, 1999, and 2001, serving as the foundation for subsequent follow-ups. A multistage stratified probability sampling approach was used, starting with the selection of postcode sectors followed by the sampling of household addresses. In wave 1 of the ELSA, 12,099 participants from 11,578 households were included. To maintain representation of younger age groups as the original cohort aged, refreshment samples were introduced in waves 3, 4, 6, 7, 9, and 10 using younger participants from the Health Survey for England [16]. Inverse probability weighting was further used to reduce bias from nonresponse and ensure that the samples were representative of the target population. Weights were calculated for participants, and participants not representative received a weight of 0 or were coded as system missing. The sociodemographic characteristics of ELSA participants are similar to those of the national census, which suggests that the ELSA sample is broadly representative of the population of England [15]. More details about the study design and weighting strategy are provided in the publicly available online ELSA user guide [17].

### Procedures

Frailty was assessed using a previously validated (in the ELSA) 31-item FI constructed using the health deficit accumulation approach [18,19]. Multiple dimensions, including self-reported health conditions, functional limitations, self-reported health status, mental health, and cognitive status, were assessed to capture the overall health status of individuals [18]. Details regarding specific items drawn to construct the FI are described in Section 1 in Multimedia Appendix 1. Participants with more than 20% data missingness for the FI were excluded [20]. The FI was computed as the ratio of present health deficits to the total potential deficits, yielding a continuous score from total fitness (0) to total frailty (1). An FI of 0.25 or higher was coded as frailty based on defined cutoff points from the study by Song et al [21]. Both continuous FI and categorical frailty were used for further analyses.

Explanatory variables from different dimensions selected based on the data availability in the ELSA and biological plausibility were used to explain the potential temporal changes in frailty and project its future prevalence. The following variables were included: age (50-64, 65-74, 75-84, and ≥85 years), sex (male and female), educational level (lower, indicating an educational level below the upper secondary level and/or having finished education before the age of 16 years; intermediate, indicating upper secondary education or a vocational degree and/or having finished education at the age of 16-18 years; and higher, indicating a bachelor’s degree or higher levels of tertiary education or having finished education at the age of 19 years or later), marital status (partnered or single), smoking (no or yes), metabolic equivalent of task (MET) score (MET hours per week were calculated to measure intensity and duration of physical activity [22]), and housing problems (the number of reported issues among 12 housing problems investigated in the ELSA, covering problems regarding space and lighting, noise, pollution, structure and maintenance, pest infestation, and temperature). Additionally, to depict the impacts of the socioeconomic development of England, the Socio-Demographic Development Index (SDI) was also used, which is based on 3 indicators associated with health outcomes: income per capita, educational attainment among individuals aged 15 years or younger, and total fertility rate among women aged under 25 years [23]. Finally, to account for effects of time and other unobserved factors, a year variable indicating studied year was also included. Detailed rationales and measurements for different variables are described in Section 2 in Multimedia Appendix 1.

### Statistical Analysis

To obtain a nationally representative estimate, all statistics were weighted [24], and only representative individuals were included in the analyses.

Characteristics of participants were presented as means and SDs for continuous variables or frequencies and percentages for categorical variables by wave. Crude prevalences of frailty across waves from 2002 to 2022 were calculated as the proportion of participants with frailty. Nationally representative estimates were then obtained by weighting the crude prevalence to account for sampling probability and differential nonresponse. The prevalence of frailty as well as the mean value of the FI from 2002 to 2022 was estimated in the overall sample and subgroups stratified by age, sex, educational level, and marital status. Finally, the number of individuals affected by frailty among the population aged 50 years and older in England was estimated based on the population size of England each year estimated by the UK Office for National Statistics (ONS) [25].

To explore the temporal trend of frailty prevalence, joinpoint regression analysis, allowing for segmenting the temporal variations by using a piecewise regression model, was used. The annual percentage change (APC) between break points and average APC (AAPC) in the entire time horizon were then presented and regarded as statistically significant under the criteria of *P*<.05 [26]. The analysis was conducted in the overall sample and subgroups by age, sex, educational level, and marital status.

On the basis of a previous study [24], a linear probability model (LPM) was used to explore the driving factors associated with the change in frailty between 2002 and 2022 using cluster-robust SEs at the individual level to account for heteroscedasticity and within-individual correlation across repeated observations. The contribution of each explanatory variable, which was the proportion of the temporal changes in frailty prevalence that it explained, was denoted by the following equation [24]:

$\beta_{j}({\bar{x}}_{j,2022}-{\bar{x}}_{j,2002})/({\bar{y}}_{2022}-{\bar{y}}_{2002})$

β_*j*_ denoted the coefficient of the explanatory variable estimated from the linear probability regression model, and ${\bar{x}}_{j2022}$ and ${\bar{x}}_{j,2002}$ were the wave averages of explanatory variables in 2022 and 2002, respectively, separately across individuals. ${\bar{y}}_{2022}$ and ${\bar{y}}_{2002}$ were the averages of frailty prevalence in 2022 and 2002, respectively, across individuals [24]. The future scenario-based prevalence of frailty in 2040 was projected using the LPM. Projected values of each explanatory variable and its corresponding model’s coefficient were plugged into the model to project changes in frailty between 2002 and 2040. Specifically, the prevalence was estimated as the weighted sum of predicted probabilities using the mean or proportion of each explanatory variable in the future population. A summary of future covariate assumptions with sources can be found in Section 3 in Multimedia Appendix 1. Age and sex structures in 2040 in England were drawn from the UK ONS [27]. Educational attainment in 2040 was assumed to keep consistent with the situation in 2022 considering that the population of interest were aged 50 years or older, the youngest people in the target population would be aged over 30 years in 2022, and most people would have already completed their education. Assumed linear trends in marital status, MET, and housing problems were applied when forecasting corresponding values in 2040. Trends in the prevalence of smoking and SDI were obtained from the Global Burden of Disease study, and the annual change rate was applied to estimate the value of smoking prevalence and SDI in 2040. Calendar year was included as a continuous linear covariate and extended to the projection years assuming a linear continuation of the unobserved effects. This was justified by the broadly linear and nonsignificant overall trend observed between 2002 and 2022. Finally, the number of individuals affected by frailty among adults aged 50 years or older in England was calculated based on projected prevalence in 2040. To project the temporal trend, the frailty prevalence was also estimated in 2024, 2028, 2032, and 2036 using the same approach. Of note, all are scenario-based projections, not predictions.

Sensitivity analyses for testing the robustness of projection were conducted following probabilistic and scenario-based methodologies. First, a nonparametric bootstrap simulation was conducted with 1000 replications to obtain the 95% CI of projected frailty prevalence in 2040. The bootstrap 95% CI was interpreted as model-only uncertainty, conditional on the assumed future covariate values. Different values of explanatory variables with various assumptions were then imputed to obtain the projected prevalence of frailty in different scenarios. Assumptions included (1) projections of the age-sex structure of the population under different scenarios (low immigration and high immigration) [27]; (2) smoking prevalence increasing or decreasing by 20% relative to the original projected values; (3) 10% and 20% more individuals of an intermediate and higher educational level; and (4) use of the lower and upper 95% CI bounds of the likelihood of being single, MET hours per week, and number of housing problems. Additionally, because frailty was analyzed as a binary outcome, LPM may exhibit heteroscedasticity and generate fitted values outside the valid probability range. A logistic regression model using the same covariates, sampling weights, and clustering specification as the primary analyses was fitted, and average marginal effects were estimated from the logistic regression model to facilitate comparison with the probability differences estimated by the LPM. To assess the potential overlap between the MET and the mobility-related items included in the FI, a sensitivity analysis in which the FI was reconstructed by excluding the mobility and physical performance deficits (items 23 to 29 in Section 1 in Multimedia Appendix 1) was performed. The LPM was then rerun using this modified FI, and the coefficient and contribution of the MET were compared with those from the primary analysis. Considering that the ELSA wave 10 coincided with the COVID-19 pandemic and the observed prevalence of frailty increased from 15.7% (1129/7195) in 2018 to 17.8% (1108/6226) in 2022, primary analyses were repeated after excluding data from wave 10. More details about the statistical analysis can be found in Section 2 in Multimedia Appendix 1. The joinpoint regression was conducted using the joinpoint trend analysis software developed by the National Cancer Institute (version 4.9.1.0). All other analyses were performed using Stata (version 18.0; StataCorp LLC). A 2-sided *P* value below .05 was considered significant for all statistical tests.

### Ethical Considerations

Ethics approvals for the ELSA were obtained from different committees. Details can be found in Section 2 in Multimedia Appendix 1. All participants provided informed consent. All data used in this secondary analysis were deidentified and publicly available; thus, no additional institutional review was required. Projections based on publicly available ONS aggregate population data do not require additional ethical approval.

## Results

After exclusions, a total of 85,190 records from 18,348 participants were included in the analysis, with a breakdown of the number of participants in each wave available in Section 4 in Multimedia Appendix 1. In the 2002 baseline survey, the mean age was 65.1 (SD 10.5) years, and 53.6% (6015/11,218) were female. A total of 59.5% (6678/11,218) had a lower educational level, and 31.4% (3522/11,218) were single. The characteristics of the participants across waves can be found in Section 5 in Multimedia Appendix 1. Of note, an improvement in educational attainment (proportion with a lower educational level from 6678/11,218, 59.5% to 1460/6226, 23.4%) was observed from 2002 to 2022. Section 6 in Multimedia Appendix 1 shows the characteristics of the included participants and those excluded for missing frailty data. Compared with included participants, excluded participants were older (mean age 70.9, SD 11.9 vs 61.9 years, SD 9.8) and more likely to report smoking (193/205, 94.1% vs 11,196/18,348, 61.0%) and had a lower mean MET score (4.8, SD 1.1 vs 5.8, SD 3.7).

Figure 1 shows the prevalence of frailty and the size of the frail population among community-dwelling adults aged 50 years and older in England from 2002 to 2022. The observed frailty prevalence in 2002 was 15.5% (1738/11,218) with a mean FI of 0.13 (SD 0.12). An increase of 2.3 percentage points in observed frailty prevalence was found in the previous 2 decades, reaching 17.8% (1108/6226) in 2022 with a corresponding mean FI of 0.14 (SD 0.13). The estimated number of individuals with frailty increased from 2,560,122 to 3,791,038 in 2022 among the community-dwelling population aged 50 years and older in England.

The prevalence of frailty across subgroups is shown in Figure 2. Those with advanced age, who were female, had a lower educational level, and/or were single had a consistently higher prevalence of frailty. In 2022, the prevalence of frailty was 42.6% (2652/6226) in the population aged 85 years and older compared to 12.9% (803/6226) in the age group of 50 to 64 years. The prevalence was 21.8% (1357/6226) among female individuals and was 13.5% (840/6226) among male individuals. Individuals with a lower educational level had a frailty prevalence of 32.1% (1998/6226), in contrast to 8.2% (510/6226) among those with higher education. Finally, frailty was more common among single individuals (1500/6226, 24.1%) than among those with partners (903/6226, 14.5%).

**Figure 1.**
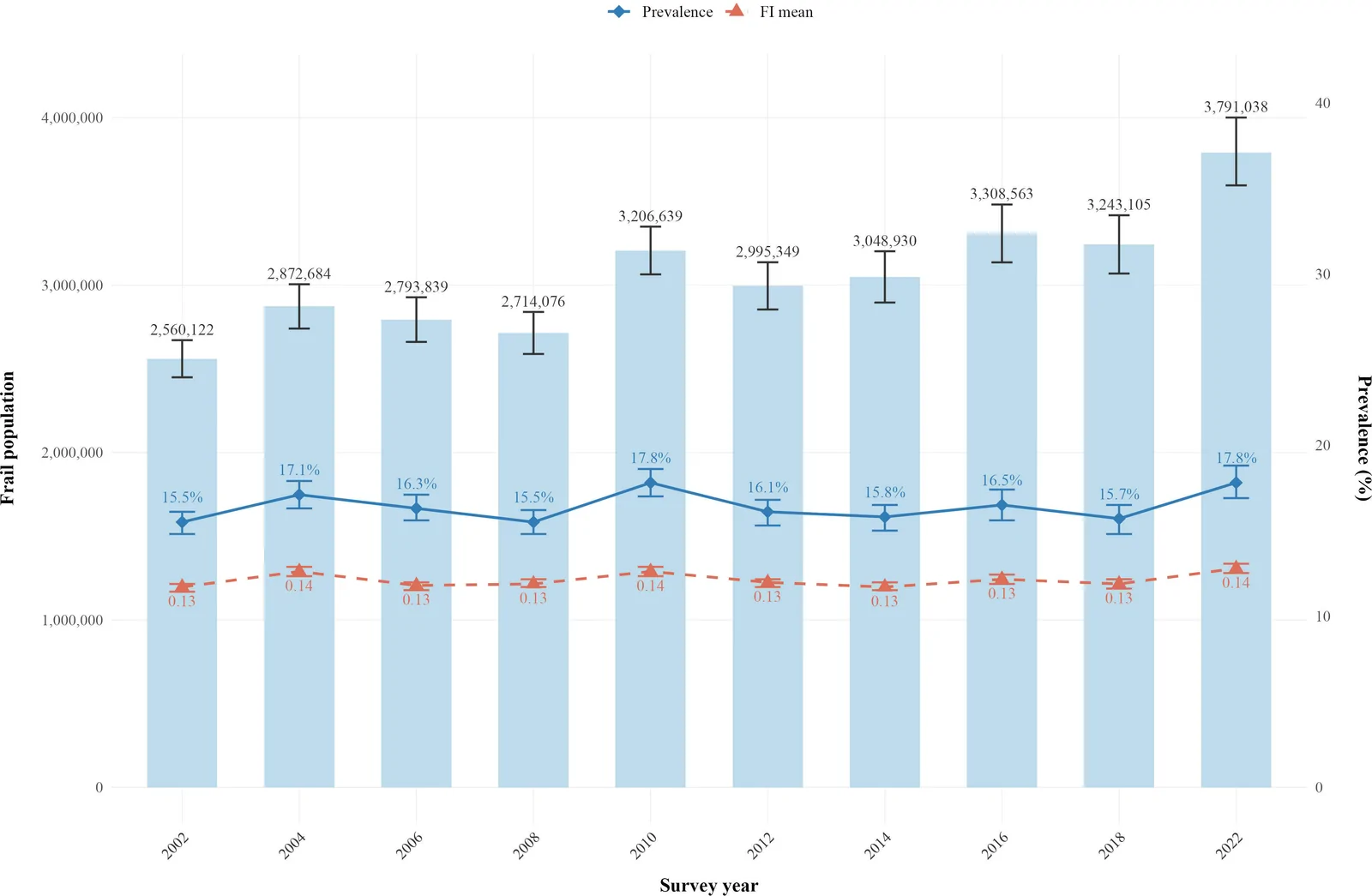
Prevalence of frailty and frail population from 2002 to 2022. FI: frailty index.

**Figure 2.**
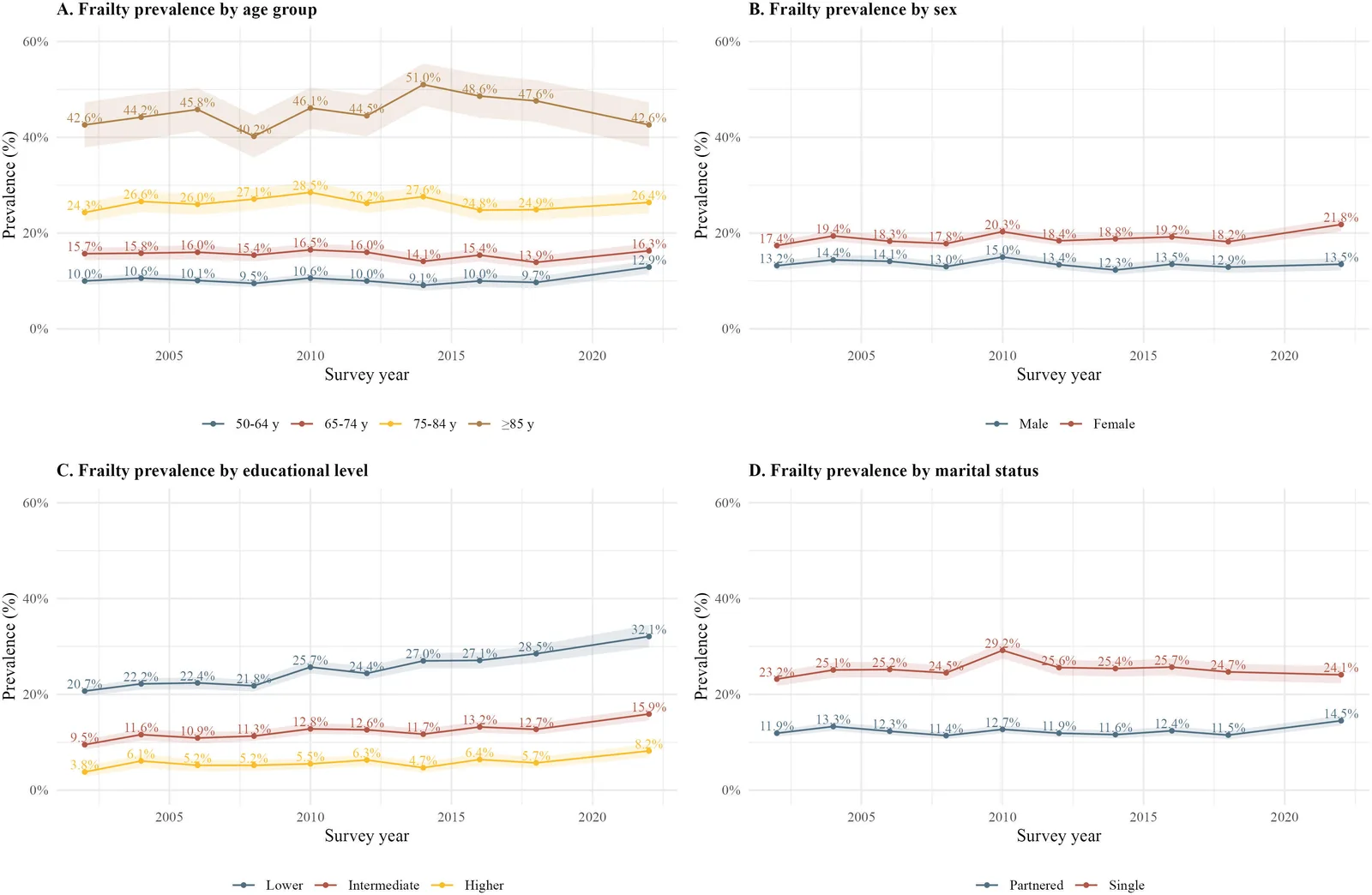
Prevalence of frailty by subgroup.

Table 1 summarizes the temporal trend of frailty prevalence. In the overall sample, the AAPC for frailty prevalence from 2002 to 2022 was 0.23% and not statistically significant, with no joinpoints identified. However, statistically significant upper trends were found among individuals with lower (AAPC=2.08%; *P*<.001) and intermediate (AAPC=1.91%; *P*=.03) educational level. Among single individuals, a significant increasing trend (APC=1.92%; *P*<.01) in frailty prevalence was observed first from 2002 to 2010, followed by a significant decreasing trend (APC=–1.13%; *P*<.05) from 2010 to 2022. In the subgroup of individuals aged 85 years and older, frailty prevalence was found to increase at a relative rate of 1.11% per year from 2002 to 2016. No significant temporal trends were observed in the sex subgroup. The plots for visualization of different temporal trends can be found in Sections 7 to 18 in Multimedia Appendix 1.

The linear probability regression analysis showed that being older, being female, having a lower educational level, being single, having a lower MET score, smoking, and having more housing problems were positively associated with frailty from 2002 to 2022 (Section 19 in Multimedia Appendix 1). Several determinants associated with declining frailty showed improvements in England between 2002 and 2022. Educational attainment improved, with the proportion of individuals attaining an intermediate level increasing from 28.9% (3239/11,218) to 52.1% (3243/6226), while those with a higher educational level increased from 11.6% (1301/11,218) to 24.5% (1523/6226). The mean MET score increased from 5.37 (SD 3.60) to 5.67 (SD 3.74). Smoking prevalence declined, with the proportion of smokers falling from 64.4% (7222/11,218) to 58.4% (3637/6226). Finally, the mean SDI increased from 0.80 to 0.86. Meanwhile, some factors contributing to frailty showed increases to different extents from 2002 to 2022. A higher proportion of single individuals (3522/11,218, 31.4% vs 2149/6226, 34.5%) was observed in 2022. There was an increase of 1.2% in the proportion of the population aged 85 years and older (Section 20 in Multimedia Appendix 1).

**Table 1.** Temporal trend of frailty prevalence.

	Prevalence of frailty, n (%)		AAPC^a^ (%; 95% CI)	*P* value	Joinpoint (prevalence in %)	APC^b^ (%; 95% CI)			
	2002	2022				Slope 1	*P* value	Slope 2	*P* value
Overall	1738 (15.5)	1108 (17.8)	0.23 (–0.67 to 1.02)	.61	No joinpoint	—^c^	—	—	—
Age (y)									
50-64	1121 (10.0)	803 (12.9)	1.02 (–0.51 to 2.60)	.19	2016 (10.0)	–0.51 (–1.78 to 0.77)	.35	4.75 (–1.38 to 11.26)	.10
65-74	1761 (15.7)	1015 (16.3)	–0.26 (–0.95 to 0.43)	.41	No joinpoint	—	—	—	—
75-84	2726 (24.3)	1643 (26.4)	0.02 (–1.03 to 1.08)	.97	2016 (24.8)	1.25 (–1.34 to 3.93)	.27	–0.8 (–2.29 to 0.72)	.23
≥85	4779 (42.6)	2652 (42.6)	0.09 (–1.49 to 1.69)	.91	2016 (48.6)	1.11 (0.47 to 4.41)	.02	–2.25 (–4.63 to 0.05)	.06
Sex									
Male	1481 (13.2)	840 (13.5)	–0.25 (–1.18 to 0.54)	.44	No joinpoint	—	—	—	—
Female	1952 (17.4)	1357 (21.8)	0.6 (–0.11 to 1.28)	.10	No joinpoint	—	—	—	—
Educational level									
Lower	2322 (20.7)	1998 (32.1)	2.08 (1.49 to 2.56)	<.001	No joinpoint	—	—	—	—
Intermediate	1065 (9.5)	990 (15.9)	1.91 (0.22 to 3.53)	.03	No joinpoint	—	—	—	—
Higher	426 (3.8)	510 (8.2)	2.35 (–0.39 to 5.14)	.08	No joinpoint	—	—	—	—
Marital status									
Partnered	1335 (11.9)	903 (14.5)	0.27 (–1.08 to 1.39)	.66	No joinpoint	—	—	—	—
Single	2602 (23.2)	1500 (24.1)	0.08 (–0.59 to 0.64)	.87	2010 (29.2)	1.92 (0.60 to 5.04)	.003	–1.13 (–3.28 to –0.28)	.01

a AAPC: average annual percentage change.

b APC: annual percentage change.

c Not applicable.

A total of 86.3% of the change in frailty prevalence was explained by this model. The overall change in frailty prevalence reflects the net result of opposing contributions from different factors, and a detailed contribution of each explanatory variable is shown in Figure 3. Aging population structure, a higher proportion of individuals being single, and more housing problems accounted for 8.7%, 6.0%, and 0.9% of the overall increase, respectively. Of note, year indicating other unobserved factors such as socioeconomic transitions, pandemic impacts, and hidden health exposures contributed to 440.6% of the increase. Conversely, improvements in sociodemographic conditions and lifestyle behaviors in England substantially mitigated the rise in frailty. The increase in SDI accounted for the largest reduction in frailty prevalence, offsetting up to 222.5% of the overall increase. Improvements in educational attainment explained 94.9% of the decrease in frailty, and more physical activity among the population aged 50 years and older contributed to a 44.8% reduction. Furthermore, a decline in smoking prevalence and fewer female individuals accounted for 6.8% and 0.9% of the decrease, respectively. Healthier lifestyle behaviors altogether offset 51.6% of the decrease. Detailed explained changes and corresponding proportions by subgroup can be found in Section 21 in Multimedia Appendix 1.

On the basis of the projected value of explanatory factors (Section 22 in Multimedia Appendix 1), the scenario-based projected prevalence of frailty among the English community-dwelling population aged 50 years and older was estimated as 20.5% in 2040, increasing from 17.1% in 2024. The corresponding projected number of individuals with frailty in England also increased from 3,829,786 in 2024 to 5,213,689 in 2040 based on extrapolating to the English population from ONS estimates on a national scale (Section 23 in Multimedia Appendix 1).

**Figure 3.**
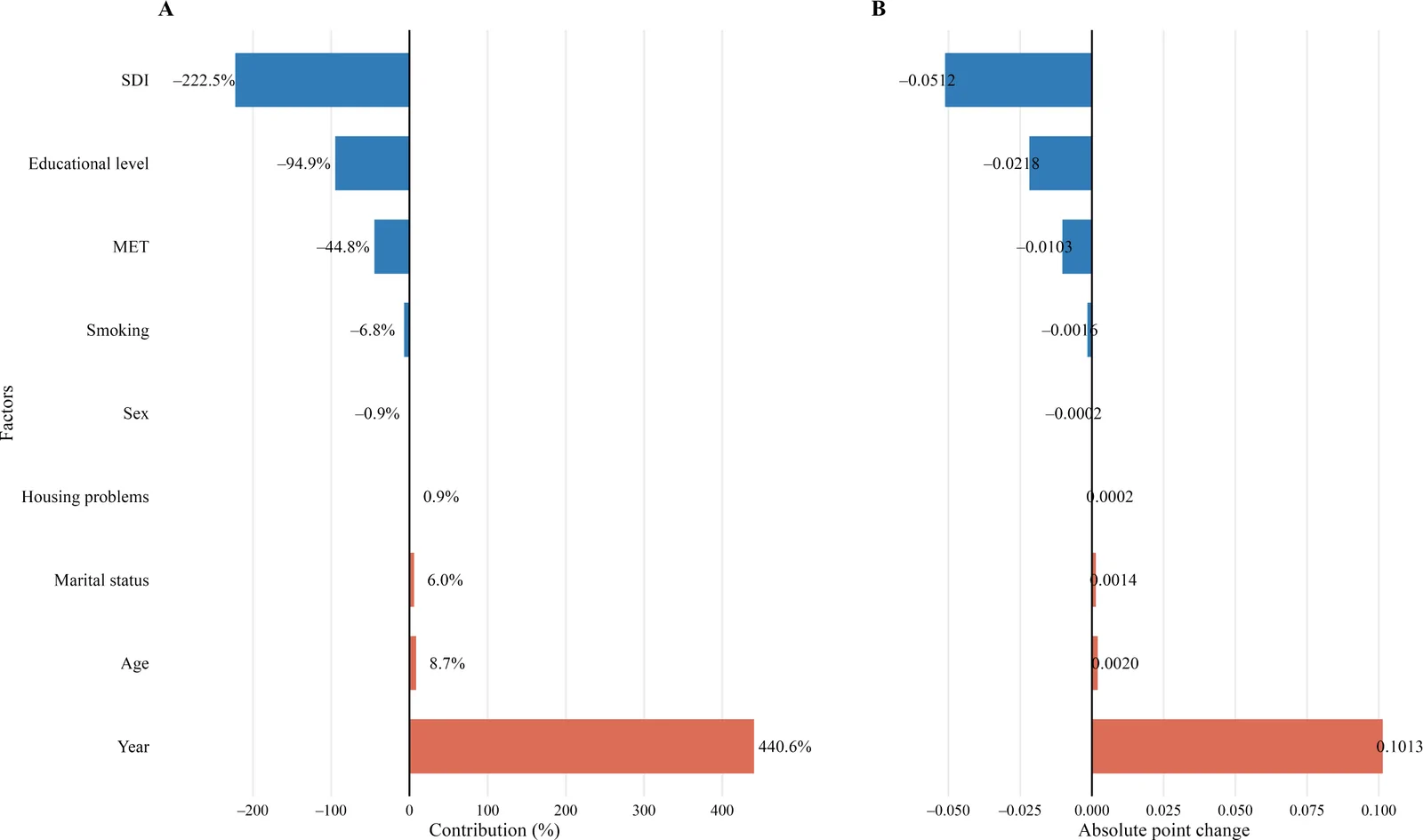
Contribution of different factors to changes in frailty prevalence in England between 2002 and 2022. Negative values indicate factors that offset the increase. Percentage contributions are relative to the net change and may exceed 100% or be negative; they do not add up to 100%. MET: metabolic equivalent of task; SDI: Socio-Demographic Development Index.

Sensitivity analyses for testing robustness of projection are shown in Figure 4. Bootstrap suggested a 95% CI of frailty prevalence in 2040 ranging from 19.2% to 21.9%, indicating a corresponding frail population size between 4,877,929 and 5,567,818. Immigration had limited effects on frailty prevalence, but the number of individuals with frailty could reach 5,357,676 under a scenario of high immigration. Assumptions of more educational improvement, fewer single individuals, more physical activity, and less smoking and housing problems were all associated with lower frailty prevalence and a lower frail population. Section 24 in Multimedia Appendix 1 shows the results estimated from the logistic regression model, showing that the direction and statistical significance of the associations were broadly consistent with those observed in the LPMs. Although some differences in the magnitude of the estimated effects were observed, particularly for the age group of 85 years and above (0.079 vs 0.176), the overall pattern of associations remained unchanged. In the sensitivity analysis excluding mobility and physical performance items from the FI, MET remained a statistically significant, independent protective factor (coefficient=–0.026; *P*<.001; Section 25 in Multimedia Appendix 1). Its contribution to offsetting the increase in frailty prevalence was 33.4% (Section 26 in Multimedia Appendix 1). In the sensitivity analysis excluding wave 10, the directions and statistical significance of all explanatory variables remained unchanged. The magnitudes of the LPM coefficients and factor contributions were also essentially unchanged (Sections 27 and 28 in Multimedia Appendix 1).

**Figure 4.**
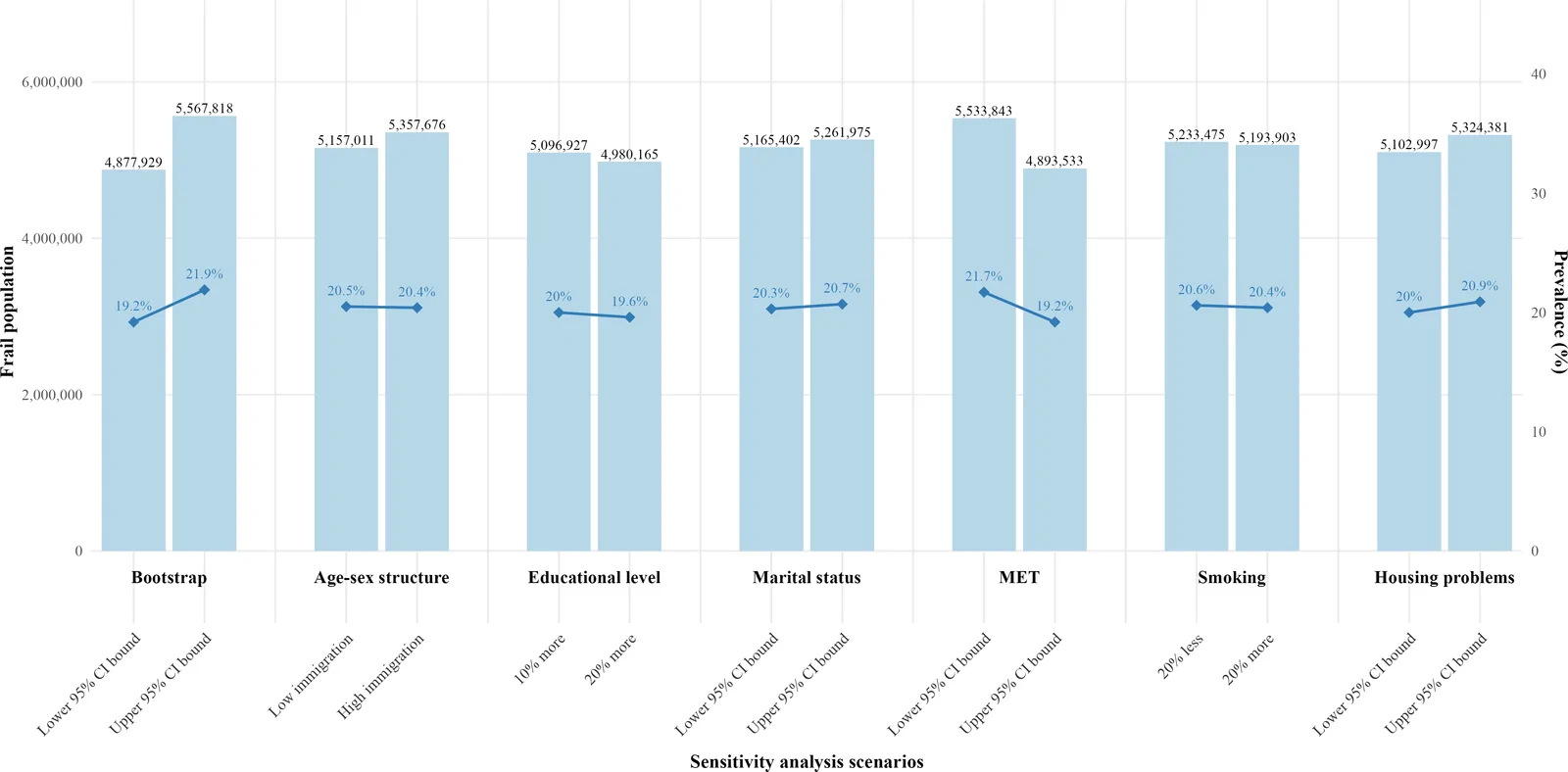
Sensitivity analyses of projected prevalence. The bootstrap 95% CI was interpreted as model-only uncertainty. MET: metabolic equivalent of task.

## Discussion

This study found that the frailty prevalence in England among the community-dwelling population aged 50 years and older was broadly stable over 20 years, with a non–statistically significant increase from 15.5% (1738/11,218) in 2002 to 17.8% (1108/6226) in 2022, with the absolute number of individuals with frailty rising from 3,791,038 to 5,213,689. The aging population structure, a growing proportion of single individuals, and more housing problems contributed to frailty prevalence. However, these factors were largely offset by sociodemographic development in England, improvement in educational attainment, adoption of healthier lifestyles including more physical activity and less smoking, and a decreasing female proportion of the population, leading to the small overall change in frailty prevalence.

Over the past 2 decades, frailty prevalence in England has shown fluctuations but remained generally stable. This is consistent with the trends observed in healthy life expectancy within the same time frame [28]. An increase in frailty prevalence from 26.5% to 38.9% between 2006 and 2017 among the community-dwelling population aged 50 years and older was reported using primary care electronic health records [10]. This higher level and steeper rise could be attributed to several factors. First, the electronic FI was applied, with different items and thresholds from those used in the present study. Second, individuals in nursing homes were included, in contrast to the community-dwelling population considered in this study. Additionally, a tendency toward overselection of less healthy individuals inherent in retrospective electronic health record–based studies might have led to an overestimation of frailty prevalence [29]. In contrast, a modest increase from 13.5% to 16.3% over 9 years (2011-2020) was reported in China, while decreasing trends have been observed in high-income countries such as Finland and South Korea [30-32]. These findings provide evidence to guide public health approaches to prevention and for policymakers and health care providers to predict population health and care needs.

There is likely to be a substantial rise in care and health care demands because of the growing frail population size [5]. Resource input and innovation may be required in the future to support adults with frailty by integrating public health approaches with wider health, social, and long-term care systems [33]. Moreover, disparities in frailty prevalence and upward trends identified in specific subgroups highlighted the vulnerable subpopulations. On the basis of the results from this study, resources could be appropriately prioritized for older adults, female or single individuals, and those with lower educational attainment. Of note, this study excluded nursing home residents, among whom frailty is more common and more severe; our prevalence and projected absolute numbers are likely conservative estimates of the total population burden. Policy inference should therefore be interpreted as relevant to community-dwelling older adults, and separate estimates or projections for care home populations are needed for whole-population care planning.

This study provided a framework to explain changes in frailty prevalence and adopt targeted interventions accordingly from the perspectives of sociodemographic development, lifestyle behavior, environmental factors, and social development. Sociodemographic development and improvements in modifiable factors were key factors curbing the rapid increase in frailty prevalence. A higher SDI, reflecting broader social and economic development, is associated with better access to education in early life, improved living conditions, and stronger health care infrastructure—all of which contribute to more favorable health outcomes [34]. The benefits of education and healthier lifestyles were widely recognized by previous studies, suggesting the importance of shifting the care model further upstream by investing more in preventive measurements [35-38]. Among these, education appeared particularly influential in our study. This might reflect the cumulative advantages of higher education, such as increased health literacy and better employment opportunities [38]. Therefore, a call on governments is needed to invest in early-life education and address the rising dropout rates in postsecondary enrollment to promote better long-term outcomes [38]. Although being single and housing problems accounted for a small proportion of the increase in prevalence, their significance in the regression model still offered insights for future interventions. Single individuals showed a higher risk of frailty, potentially due to increased psychological and emotional stress and reduced social support, highlighting the need for targeted interventions [39]. Additionally, housing is gradually gaining institutional recognition for its role as a social, economic, environmental, and cultural determinant of health, and its impacts on frailty have also been established [40,41]. Despite minor changes in housing problems during the study period, the cost of living crisis since 2020 might significantly worsen housing conditions and increase frailty risk, warranting closer attention [42].

This study has some limitations. First, the measurement of frailty relied on self-reported items in the ELSA, which might bring recall or response biases into the analysis. This was mitigated to some extent by the use of a standardized protocol for the ELSA, meaning that responses across waves should be reliable and comparable. Second, nursing home individuals were not included in this study; thus, the estimate of frailty prevalence should be generalized with caution to the full population. Third, although 86.3% of the changes in frailty prevalence were explained, the study year accounted for the largest proportion of the observed increase. However, as a residual proxy for unmeasured effects, study year may have masked the specific drivers of the rising frailty prevalence, such as socioeconomic transitions, pandemic impacts, and hidden health exposures. Consequently, key contributors to this trend may not have been fully identified. Additionally, to balance the simplicity and effectiveness of the projection model, and due to limitations in data availability or substantial missingness in self-completed questions in the ELSA, several variables known to influence frailty, such as nutrition, alcohol consumption, and social isolation, were not included in the analysis. Moreover, LPM may generate fitted values outside the valid probability range (0-1) and commonly exhibit heteroscedasticity. In our analysis, 20.7% of fitted values were below 0, although none exceeded 1. However, the use of cluster-robust SEs at the individual level partially addressed the issue of heteroscedasticity, and the sensitivity analysis using a logistic regression model confirmed the robustness of the results. Although analyses applied the ELSA survey weights to improve representativeness, weighting can only partially account for observed differences between eligible and noneligible respondents. Consequently, the estimates may not fully represent the entire community-dwelling population aged 50 years and older in England. Additionally, the bootstrap CI captures only model uncertainty, while scenario analyses vary assumptions individually and do not jointly account for uncertainty in future covariates, coefficients, baseline prevalence, and model structure. A fully joint uncertainty assessment is needed for future research. Finally, the mechanisms underlying the associations between these factors and frailty were not explored and may warrant future research to inform more targeted interventions. These limitations are balanced by key innovations, including 20 years of nationally representative data; a comprehensive set of explanatory variables; and an innovative analytical framework that combines temporal trend estimation, population-level decomposition, and scenario-based projection to 2040. Future studies are warranted to explore frailty prevalence in the entire population, incorporate objective frailty measurements, and identify unobserved driving factors for increasing frailty prevalence.

In conclusion, the past 20 years have seen a statistically nonsignificant increase in frailty prevalence among the community-dwelling population aged 50 years and older, and sociodemographic development, improvement in educational attainment, and adoption of healthier lifestyles served as the main curbing factors. By 2040, the scenario-based projected frailty prevalence is expected to increase modestly but with substantial growth in frail population size. Enhancing educational attainment and embedding healthy lifestyle behaviors across the population are suggested as key addressing strategies.

## Supplementary material

10.2196/98665 Multimedia Appendix 1 Supplementary methodology details and results.

## Abbreviations

AAPC: average annual percentage change
APC: annual percentage change
ELSA: English Longitudinal Study of Ageing
FI: frailty index
LPM: linear probability model
MET: metabolic equivalent of task
ONS: Office for National Statistics
SDI: Socio-Demographic Development Index
