# Supplementary material for: Temporal Trends in the Prevalence of Frailty in England From 2002 to 2022, With Forecasts to 2040: Longitudinal Secondary Analysis of a National Cohort Study
Source: JMIR Aging. 2026 Nov 3;9:e98665. doi: 10.2196/98665 (PMC13730213; doi:10.2196/98665)
Supplement: Supplementary file 1 [file aging-v9-e98665-s001.pdf]

Supplementary materials

The prevalence of frailty in England from 2002 to 2022 and future forecasts to 2040: a longitudinal national cohort study

Table of Contents

Section 1: Detailed items and calculation of FI 3
Section 2: Supplementary methodology 5
Explanatory variables 5
Statistical analysis 5
Ethic approval 6
Section 3: Summary of future covariate assumptions with source 7
Section 4: Flowchart of sample inclusion in each wave 8
Section 5: Characteristics of participants in each wave 9
Section 6: Characteristics of included and excluded sample for missing FI data 10
Section 7: Joinpoints in the overall sample 11
Section 8: Joinpoints in the subgroup of the population aged 50-64 12
Section 9: Joinpoints in the subgroup of the population aged 65-74 13
Section 10: Joinpoints in the subgroup of the population aged 75-84 14
Section 11: Joinpoints in the subgroup of the population aged ≥85 15
Section 12: Joinpoints in the female subgroup 16
Section 13: Joinpoints in the male subgroup 17
Section 14: Joinpoints in the subgroup of lower education level 18
Section 15: Joinpoints in the subgroup of intermediate education level 19
Section 16: Joinpoints in the subgroup of higher education level 20
Section 17: Joinpoints in the subgroup of being single 21
Section 18: Joinpoints in the subgroup of being partnered 22
Section 19: Associations of explanatory factors with frailty 23
Section 20: Changes of explanatory factors between 2002 and 2022 24
Section 21: Contribution of different factors to changes in frailty prevalence in England between 2002 and 2022 25
Section 22: Projected prevalence of frailty and frail population in England in 2040 26
Section 23: Projected prevalence of frailty from 2024 to 2040 27
Section 24: Associations of explanatory factors with frailty using logistic regression model 28
Section 25: Associations of explanatory factors with frailty using modified FI 29
Section 26: Contribution of different factors to changes in frailty prevalence in England between 2002 and 2022 using modified FI 30
Section 27: Associations of explanatory factors with frailty after excluding wave 10 31
Section 28: Contribution of different factors to changes in frailty prevalence in England between 2002 and 2018 32
Section 29: Frailty prevalence with 95% CI by year, overall and by subgroup 33
Reference 34

Section 1: Detailed items and calculation of FI

No	Description of the item	Cut-off value
1	Self-reported physician diagnosed hypertension	Yes = 1, No = 0
2	Self-reported physician diagnosed diabetes	Yes = 1, No = 0
3	Self-reported physician diagnosed cancer	Yes = 1, No = 0
4	Self-reported physician diagnosed arthritis	Yes = 1, No = 0
5	Self-reported physician diagnosed chronic lung disease	Yes = 1, No = 0
6	Self-reported physician diagnosed any emotional, nervous, or psychiatric problems	Yes = 1, No = 0
7	Self-reported physician diagnosed heart attack	Yes = 1, No = 0
8	Self-reported physician diagnosed angina	Yes = 1, No = 0
9	Self-reported physician diagnosed heart failure	Yes = 1, No = 0
10	Self-reported physician diagnosed stroke	Yes = 1, No = 0
11	Self-reported physician diagnosed Parkinson's disease	Yes = 1, No = 0
12	Self-reported physician diagnosed Dementia/Alzheimer's disease	Yes = 1, No = 0
13	Self-reported physician diagnosed general health status	Very poor = 1, Poor =0·75, Fair = 0·5, Good =0·25, Very good = 0
14	Difficulty with dressing	Yes = 1, No = 0
15	Difficulty with bathing or showering	Yes = 1, No = 0
16	Difficulty with eating	Yes = 1, No = 0
17	Difficulty with getting in and out of bed	Yes = 1, No = 0
18	Difficulty with using the toilet	Yes = 1, No = 0
19	Difficulty with managing money	Yes = 1, No = 0
20	Difficulty with taking medication	Yes = 1, No = 0
21	Difficulty with shopping for groceries	Yes = 1, No = 0
22	Difficulty with preparing meals	Yes = 1, No = 0
23	Mobility: difficulty with walking 100 yards or one block	Yes = 1, No = 0
24	Mobility: difficulty with getting up from a chair after sitting for long periods	Yes = 1, No = 0
25	Mobility: difficulty with climbing several flights of stairs without resting	Yes = 1, No = 0

26	Mobility: difficulty with lifting or carrying weights over 10 pounds/jins	Yes = 1, No = 0
27	Mobility: difficulty with picking up a coin from the table	Yes = 1, No = 0
28	Mobility: difficulty with stooping, kneeling, or crouching	Yes = 1, No = 0
29	Mobility: difficulty with reaching arms above shoulder level	Yes = 1, No = 0
30	Depression: CESD-8 questionnaire	CESD-8 $\geq 4 = 1$, $<4 = 0$ in the ELSA
31	Cognition score: (memory test score + orientation test score) / 14	1-cognition score (continuous, ranging from 0 to 1)

Section 2: Supplementary methodology

Explanatory variables

Explanatory variables from different dimensions selected based on the data availability in ELSA and biological plausibility were used to explain the potential temporal changes in frailty and project the future prevalence of frailty. A recent large cohort study including 469 301 participants from UK Biobank evaluated 90 risk factors for frailty across ten domains and found health and medical history, psychosocial factors, physical activity, and sociodemographic explained the most variation in frailty, accounting for 11·9%, 10·4%, 10·1%, 9·7% respectively [main text reference 37]. Considering the overlapping with FI items, health and medical history were not considered. Incorporating other existing evidence, following explanatory variables were included: sociodemographic including age, sex, marital status, and education, lifestyle factors including physical activity, and smoking [main text reference 10, 35-37]. Since there is more and more institutional recognition of the role of housing as a determinant of health, and WHO also defined it as an intermediary determinant when framing for actions on social determinants of health [main text reference 41], housing problems was also included as an explanatory variable. At last, to depict the impacts of socioeconomic development of England, socio-demographic development index (SDI) was included. SDI can be used to assess the overall social and economic development level within a country based on three indicators associated with health outcomes: income per capita, educational attainment among individuals aged 15 years or younger, and total fertility rate among women aged under 25 years [main text reference 23]. SDI for England in each year could be obtained from Global Burden of Diseases (GBD). At last, to account for effects of time and other unobserved factors, a year variable indicating studied year was also included.

Sex (male vs female) and age were self-reported. Age was grouped into four groups based on previous study (50-64;65-74;75-84;≥85) [main text reference 10].Following previous studies, education was grouped as three levels: lower, intermediate, and higher levels of education, drawing on information about the participants' highest levels of education and age at finishing education [1]. Those having an education level of below the upper secondary level and/or finished education before the age of 16 were defined as lower education. Groups of holding upper secondary education or vocational degrees and/or finishing education at the age of 16-18 years were classified as intermediate education. Higher levels of education was defined as having a BA degree or higher levels of tertiary education or in cases of missing data, completing education at age 19 or later [1]. Marital status was defined as partnered or single. Those being unmarried or not cohabiting were defined as single, otherwise were defined as partnered.

Physical activity was defined based on the validated questionnaire in ELSA regarding the frequency of vigorous, moderate and mild physical activity (more than once per week, once per week, one to three times per month, or hardly ever) [2,3]. Additionally, the questionnaire was also validated by using objective measurements and results showed moderate correlation in moderate and vigorous activities between self-report and objectively-assessed approaches [4]. A continuous physical activity variable was constructed by applying a metabolic equivalent (MET) score to different intensity of activity and multiplying by corresponding frequency per week [main text reference 22]. Vigorous, moderate, and mild activity was assigned a MET score of 9·0, 4·5, and 2·0, respectively. Based on previous study, different self-reported frequencies were assumed or coded as 1·5 (more than once a week), 1·0 (once a week), 0·5 (one to three times a week), and 0·0 (never or hardly ever) [main text reference 22]. Each activity was assumed to be 0·5 h long [main text reference 22]. MET hours per week for different physical activities was calculated by multiplying MET of different activity, frequency, and 0·5 hours. The total MET hours per week was the sum of MET hours per week of vigorous, moderate, and mild activities. Smoking was dichotomized as yes or no according to whether ever smoked.

Housing problems were assessed by the number of accommodation problems investigated in ELSA. Participants were asked about whether his or her accommodation had following problems: 1: Shortage of space. 2: Noise from neighbours 3: Other street noise, such as traffic, businesses, factories. 4: Too dark, not enough light. 5: Pollution, grime or other environmental problems caused by traffic or industry. 6: Rising damp in floors and walls. 7: Water getting in from roof, gutters or windows. 8: Bad condensation problem. 9: Problems with electrical wiring or plumbing. 10: General rot and decay. 11: Problems with insects, mice or rats. 12: Too cold in winter. Then, the total number of housing problems was calculated for each participant.

Statistical analysis

To obtain the nationally representative estimate, all statistics were weighted.

Characteristics of participants were presented as mean and standard deviation (SD) for continuous variables or frequency and percentage for categorical variables by each wave. Crude prevalences of frailty across waves from 2002 to 2022 were calculated as the proportion of participants with frailty (FI≥0·25). Nationally representative estimates were weighted to account for sampling probability and differential non-response. The prevalence of frailty as well as mean value of FI from 2002 to 2022 was estimated in the overall sample and subgroups stratified by age, sex, education, marital status. At last, the number of individuals affected by frailty among population aged 50 years and older in England was also estimated based on the population size of England each year estimated by Office for National Statistics (ONS) in UK [main text reference 25].

To explore the temporal trend of frailty prevalence, joinpoint regression analysis, allowing for segmenting the longitudinal variations by utilizing a piecewise regression model was used. It consists of several linear segments to describe changes in trend [5]. Using log-linear modeling and the grid search method (GSM), it explored breakpoints in intervals through creating a "grid" of all possible locations for joinpoints specified by the settings and calculating the sum of the squared error (SSE) at each one to find the best possible fit. Subsequently, a Monte Carlo permutation test would employed to determine the optimal model for joinpoint regression, considering potential breakpoints ranging from 0 to 5 [6]. Then the annual percentage change (APC) between breakpoints and average annual percentage change (AAPC) in the whole time horizon were presented and regarded as statistically significant under the criteria $p < 0{\cdot}05$ [main text reference 26]. This is a simple and flexible method to facilitate identification of each key trend turning point during a long-term horizon [6]. The analysis was conducted in the overall sample and subgroups of age, sex, education, and marital status. The analysis was performed using the Joinpoint Trend Analysis Software developed by the National Cancer Institute (version 4.9.1.0, https://surveillance.cancer.gov/joinpoint/).

Based on previous study [main text reference 24], a linear probability model was used to explore the driving factors associated with the change in frailty between 2002 and 2022:

$$y_{it} = \beta_0 + \sum_{j=1}^{k} \beta_j x_{ijt} + u_{it} \quad (1)$$

In the model: "y_{it}" denoted the dummy variable of frailty for individual i at time t. K was the number of explanatory variables included in this study. "x_{ijt}" was the explanatory variable j of individual "i" at time "t". β_j represented for the coefficient of explanatory variable "x_j". "u_{it}" was

the error term. Explanatory variables in this study included age (categorial), sex, education, marital status, physical activity, smoking, housing problems, SDI, and wave year. By using the model, SEs were estimated by clustering the sample at the individual level to solve the issue of within-individual correlations. The proportion of each explanatory variable in explaining the temporal changes of frailty prevalence was denoted by [main text reference 24]:

$$\beta_j(\bar{x}_{j,2022} - \bar{x}_{j,2002})/(\bar{y}_{2022} - \bar{y}_{2002})$$

β_j denoted the coefficient of the explanatory variable estimated from the linear probability regression model, and $\bar{x}_{j2022}$, $\bar{x}_{j,2002}$ were the wave averages of explanatory variables in 2022 and 2002 separately across individuals. $\bar{y}_{2022}$, $\bar{y}_{2002}$ were the averages of frailty prevalence in 2022 and 2002 across individuals [main text reference 24].

Sensitivity analyses were conducted in the ways of and probabilistic analysis and scenarios analysis. First, a nonparametric bootstrap simulation was conducted with 1000 replications to obtain the 95% CI of projected frailty prevalence in 2040. Bootstrap is a resampling method that allows estimating the uncertainty of a statistic without making strong parametric assumptions. By repeatedly drawing samples with replacement from the observed dataset, which is assumed to be representative of the population, different "bootstrap samples" could be obtained and used to recalculate the statistic of interest, and the variability across these estimates provides a way to construct confidence intervals .Then, different values of explanatory variables under various assumptions were considered to obtain the projected prevalence of frailty in different scenarios:

1. Projections of age-sex structure of the population under two scenarios (low immigration and high immigration) were applied [main text reference 27]. Other explanatory factors were kept constant with original predictions.
2. Assumption on Smoking prevalence using two scenarios: increase or decrease by 20% relative to the original projected values respectively. Other explanatory factors were kept constant with original predictions.
3. 10% and 20% more individuals in intermediate and higher education level. Other explanatory factors were kept constant with original predictions.
4. Lower 95% CI and upper 95% CI of prevalence of being single, with other explanatory factors being kept constant with original predictions.
5. Lower 95% CI and upper 95% CI of MET hours per week, with other explanatory factors being kept constant with original predictions.
6. Lower 95% CI and upper 95% CI of number of housing problems were used, with other explanatory factors being kept constant with original predictions.

Ethic approval

ELSA Wave 1 received ethical approval from the London Multi-Centre Research Ethics Committee on 7th February 2002 (MREC/01/2/91); Wave 2 received ethical approval from the London Multi-Centre Research Ethics Committee on 12th August 2004 (MREC/04/2/006); ELSA Wave 3 received ethical approval from the London Multi-Centre Research Ethics Committee on 27th October 2005 (05/MRE02/63); ELSA Wave 4 received ethical approval from the National Hospital for Neurology and Neurosurgery & Institute of Neurology Joint Research Ethics Committee on 12th October 2007 (07/H0716/48); ELSA Wave 5 received ethical approval from the Berkshire Research Ethics Committee on 21st December 2009 (09/H0505/124); ELSA Wave 6 received ethical approval from the NRES Committee South Central - Berkshire on 28th November 2012 (11/SC/0374); ELSA Wave 7 received ethical approval from the NRES Committee South Central - Berkshire on 28th November 2013 (13/SC/0532); ELSA Wave 8 received ethical approval from the South Central – Berkshire Research Ethics Committee on 23rd September 2015 (15/SC/0526); ELSA Wave 9 received ethical approval from the South Central – Berkshire Research Ethics Committee on 10th May 2018 (17/SC/0588); ELSA Wave 10 received ethical approval from the South Central – Berkshire Research Ethics Committee on 22nd March 2021 (21/SC/0030).

Section 3: Summary of future covariate assumptions with source

Covariate	Future covariates assumption	Source
Age and sex structure	ONS 2022-based national population projections; low- and high-immigration scenarios	ONS
Education	Held constant at ELSA 2022 distribution, because the target population has largely completed education	ELSA
Marital status	Linear extrapolation from 2002–2022 ELSA trends	ELSA
MET	Linear extrapolation from 2002–2022 ELSA trends	ELSA
Smoking	GBD England annual change rate applied to 2040	GBD
Housing problems	Linear extrapolation from ELSA trends	ELSA
SDI	GBD England annual change rate applied to 2040	GBD
Calendar year	Continuous linear covariate extended to 2040; justified by broadly linear, non-significant 2002–2022 trend	Study assumption

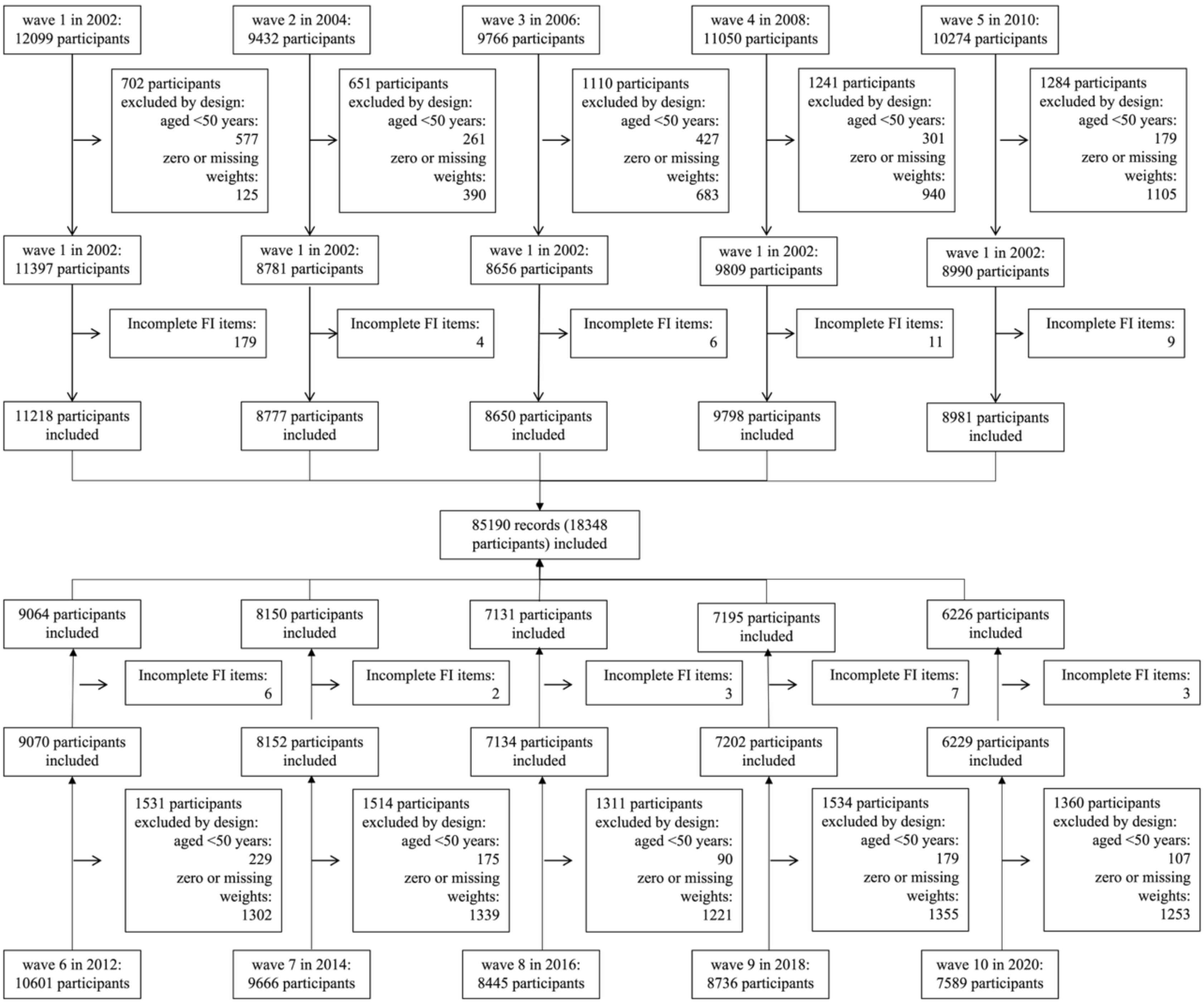

Section 4: Flowchart of sample inclusion in each wave

Section 5: Characteristics of participants in each wave

	2002: 11218		2024: 8777		2006: 8650		2008: 9798		2010: 8981		2012: 9064		2014: 8150		2016: 7131		2018: 7195		2022: 6226	
	N/Mean	%/SD	N/Mean	%/SD	N/Mean	%/SD	N/Mean	%/SD	N/Mean	%/SD	N/Mean	%/SD	N/Mean	%/SD	N/Mean	%/SD	N/Mean	%/SD	N/Mean	%/SD
Age	65·1	10·5	66·9	10·2	65·7	10·5	65·6	10·2	67·3	9·7	65·4	10·5	65·4	10·6	66·6	10·2	65·6	10·9	65·9	10·9
50-64	5878	52·4%	4134	47·1%	4491	51·9%	5259	53·7%	4234	47·1%	4733	52·2%	4196	51·5%	3373	47·3%	3637	50·6%	3216	51·6%
65-74	2911	26·0%	2434	27·7%	2194	25·4%	2429	24·8%	2566	28·6%	2384	26·3%	2201	27·0%	2117	29·7%	1960	27·2%	1584	25·4%
75-84	1895	16·9%	1707	19·5%	1469	17·0%	1553	15·9%	1575	17·5%	1435	15·8%	1289	15·8%	1197	16·8%	1143	15·9%	1058	17·0%
≥85	534	4·8%	502	5·7%	497	5·7%	557	5·7%	605	6·7%	512	5·6%	464	5·7%	444	6·2%	454	6·3%	368	5·9%
Sex																				
Male	5203	46·4%	4047	46·1%	4054	46·9%	4602	47·0%	4208	46·9%	4300	47·4%	3878	47·6%	3395	47·6%	3420	47·5%	2970	47·7%
Female	6015	53·6%	4730	53·9%	4596	53·1%	5196	53·0%	4773	53·1%	4764	52·6%	4272	52·4%	3736	52·4%	3775	52·5%	3256	52·3%
Education																				
Lower	6678	59·5%	5130	58·4%	4692	54·2%	4755	48·5%	4260	47·4%	3514	38·8%	2861	35·1%	2363	33·1%	2039	28·3%	1460	23·4%
Intermediate	3239	28·9%	2588	29·5%	2779	32·1%	3565	36·4%	3315	36·9%	3918	43·2%	3756	46·1%	3411	47·8%	3577	49·7%	3243	52·1%
High	1301	11·6%	1060	12·1%	1178	13·6%	1477	15·1%	1406	15·7%	1633	18·0%	1533	18·8%	1357	19·0%	1579	21·9%	1523	24·5%
MET	5·37	3·60	5·46	3·51	5·49	3·61	5·46	3·66	5·46	3·65	5·70	3·74	5·77	3·73	5·75	3·71	5·88	3·84	5·67	3·74
Marital status																				
Partnered	7696	68·6%	5953	67·8%	5945	68·7%	6743	68·8%	6204	69·1%	6296	69·5%	5698	69·9%	4935	69·2%	4920	68·4%	4077	65·5%
Single	3522	31·4%	2824	32·2%	2705	31·3%	3055	31·2%	2777	30·9%	2768	30·5%	2452	30·1%	2196	30·8%	2275	31·6%	2149	34·5%
Smoking																				
No	3996	35·6%	3169	36·1%	3230	37·3%	3759	38·4%	3290	36·6%	3441	38·0%	3139	38·5%	2754	38·6%	2944	40·9%	2589	41·6%
Yes	7222	64·4%	5608	63·9%	5420	62·7%	6039	61·6%	5691	63·4%	5623	62·0%	5011	61·5%	4377	61·4%	4251	59·1%	3637	58·4%
Housing problem	0·56	1·02	0·46	0·91	0·49	0·95	0·49	0·98	0·48	0·95	0·53	1·02	0·51	1·02	0·52	1·07	0·55	1·02	0·57	1·08

Section 6: Characteristics of included and excluded sample for missing FI data

		Total (N=18553)	
		Excluded sample for missign FI (N=205)	Included sample (18348)
Age (Mean, SD)		70.9 (11.9)	61.9 (9.8)
Sex			
	Male	99 (48.3%)	8,271 (45.1%)
	Female	106 (51.7%)	10,077 (54.9%)
Education			
	Lower	136 (66.3%)	8,303 (45.3%)
	Intermediate	53 (28.9%)	6,935 (37.8%)
	High	16 (7.8%)	3,110 (17.0%)
MET		4.8 (1.1)	5.8 (3.7)
Marital status			
	Partnered	142 (69.3%)	12,914 (70.4%)
	Single	63 (30.7%)	5,434 (29.6%)
Smoking			
	No	12 (5.9%)	7,152 (39.0%)
	Yes	193 (94.1%)	11,196 (61.0%)
Housing problem		0.47 (0.89)	0.60 (1.07)

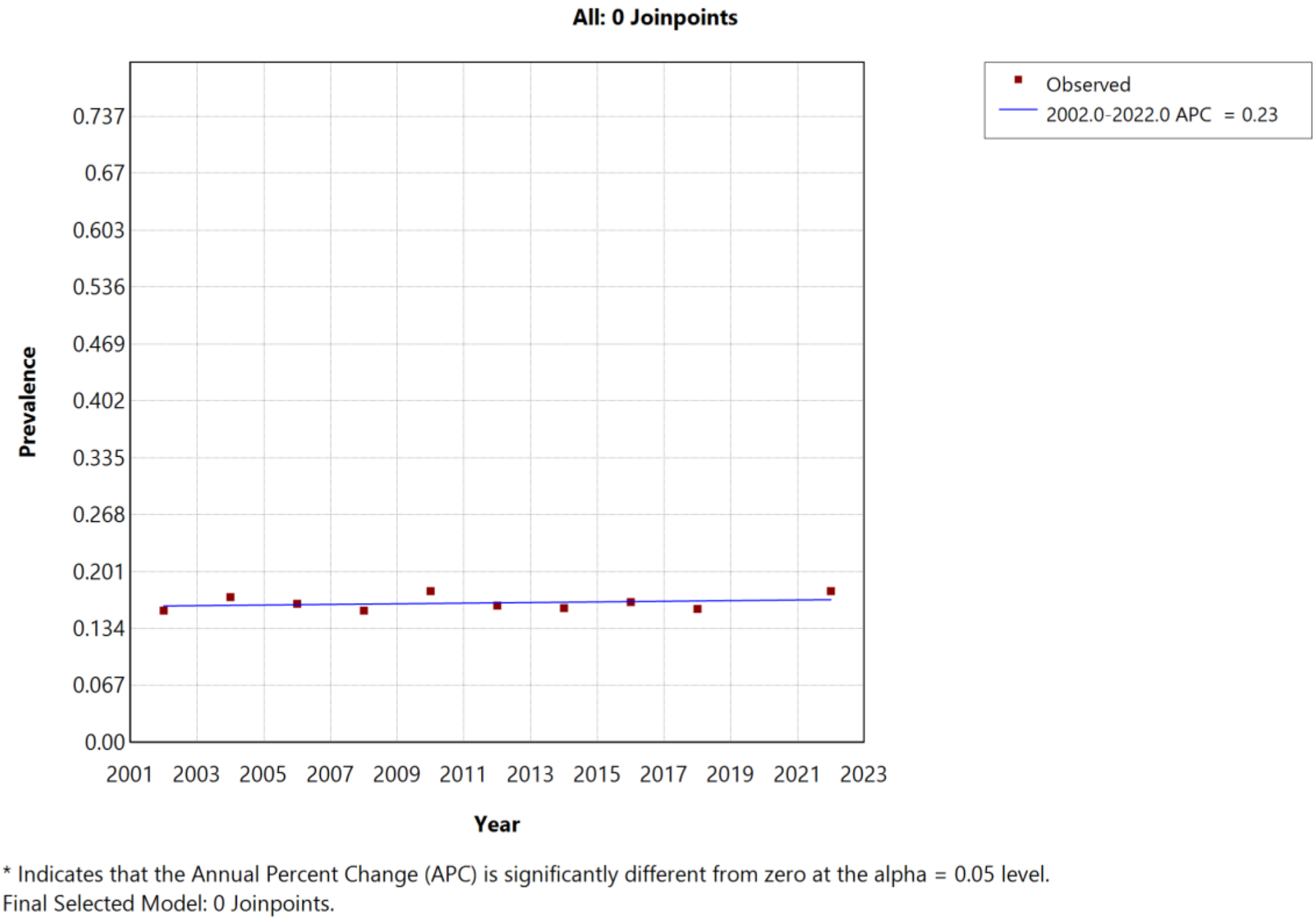

Section 7: Joinpoints in the overall sample

Note. The observed prevalence values and their 95% confidence intervals are provided in Section 29.

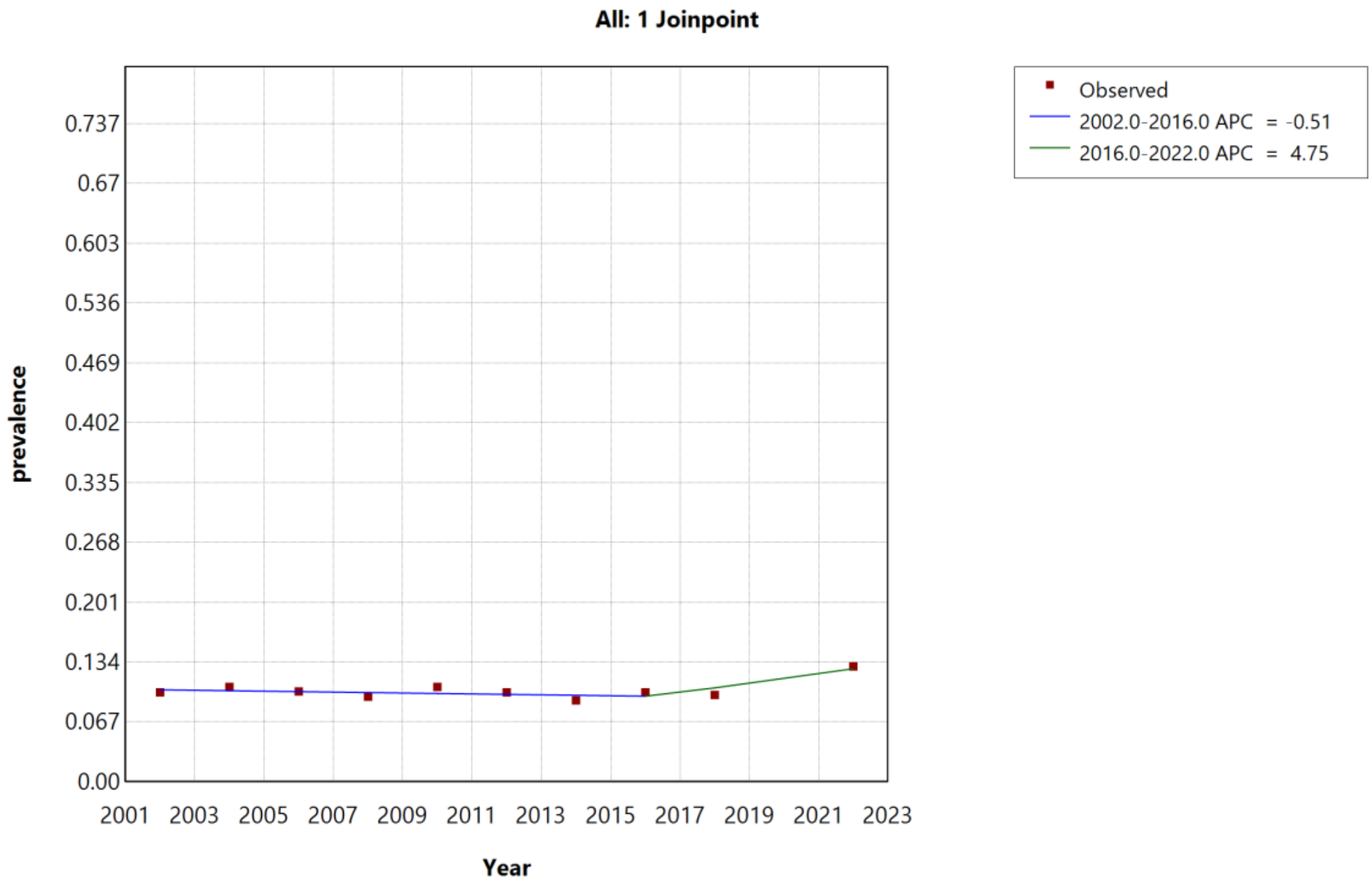

* Indicates that the Annual Percent Change (APC) is significantly different from zero at the alpha = 0.05 level.
Final Selected Model: 1 Joinpoint.

Section 8: Joinpoints in the subgroup of the population aged 50-64

Note. The observed prevalence values and their 95% confidence intervals are provided in Section 29.

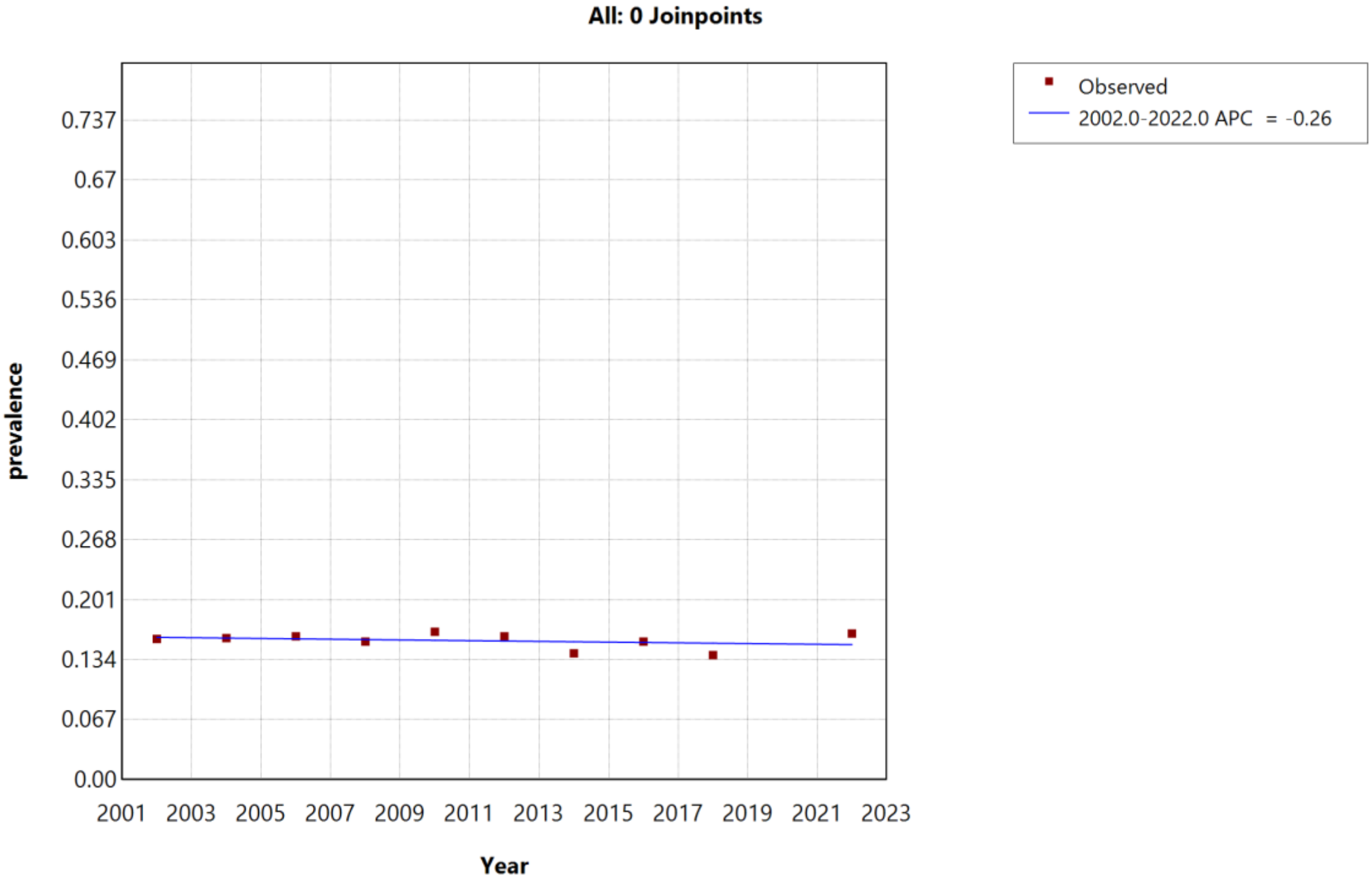

* Indicates that the Annual Percent Change (APC) is significantly different from zero at the alpha = 0.05 level.
Final Selected Model: 0 Joinpoints.

Section 9: Joinpoints in the subgroup of the population aged 65-74
Note. The observed prevalence values and their 95% confidence intervals are provided in Section 29.

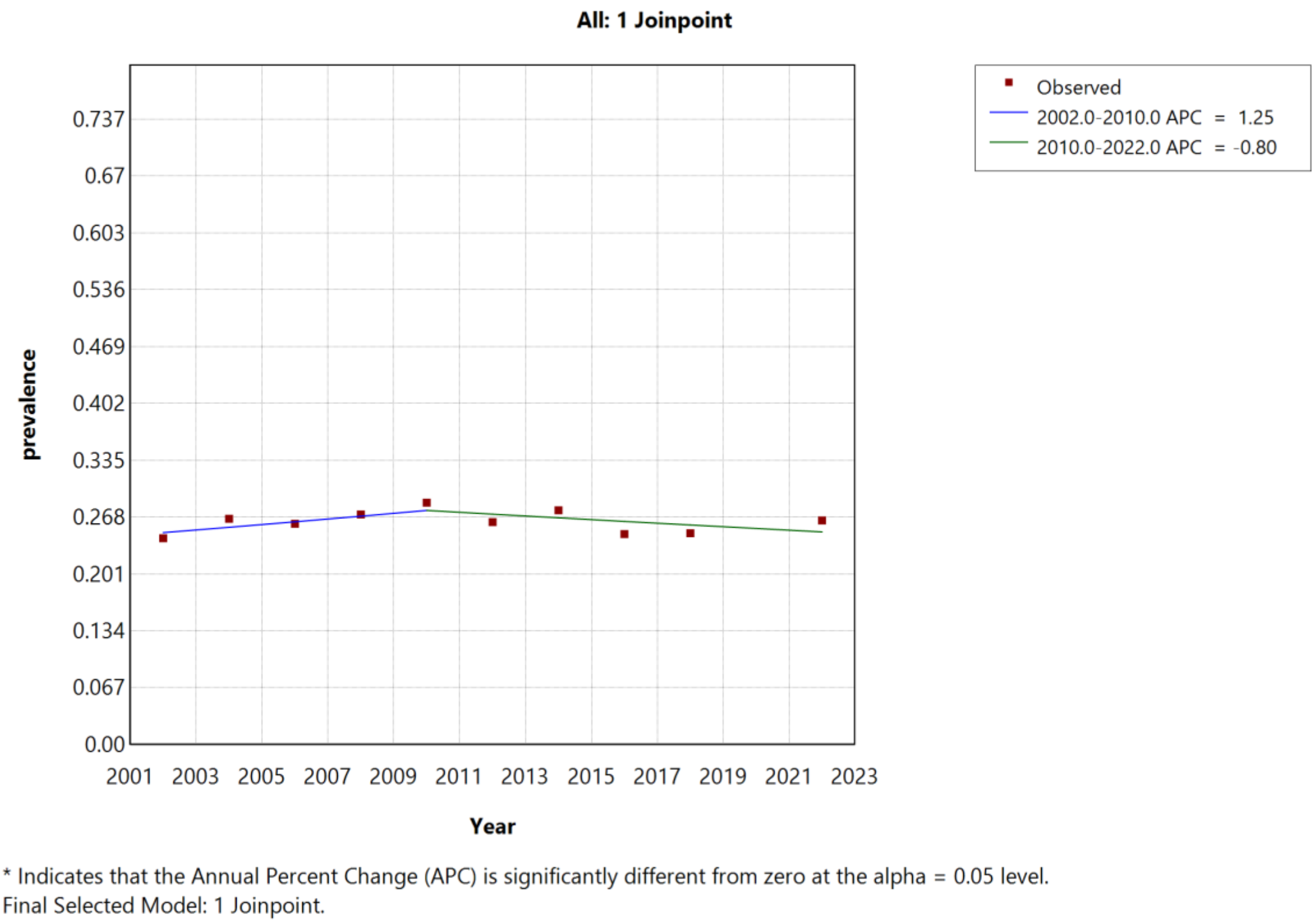

Section 10: Joinpoints in the subgroup of the population aged 75-84
Note. The observed prevalence values and their 95% confidence intervals are provided in Section 29.

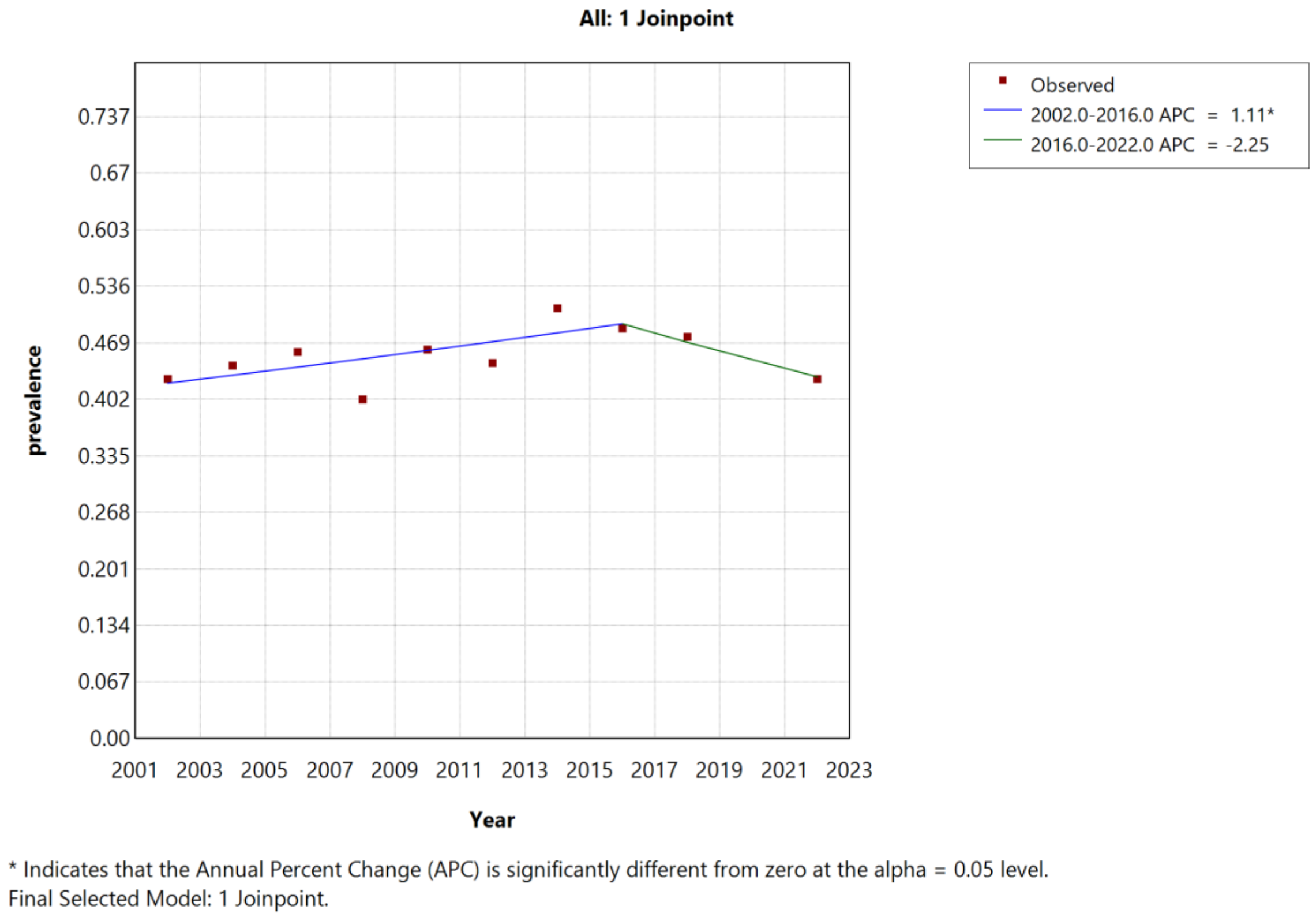

Section 11: Joinpoints in the subgroup of the population aged ≥85

Note. The observed prevalence values and their 95% confidence intervals are provided in Section 29.

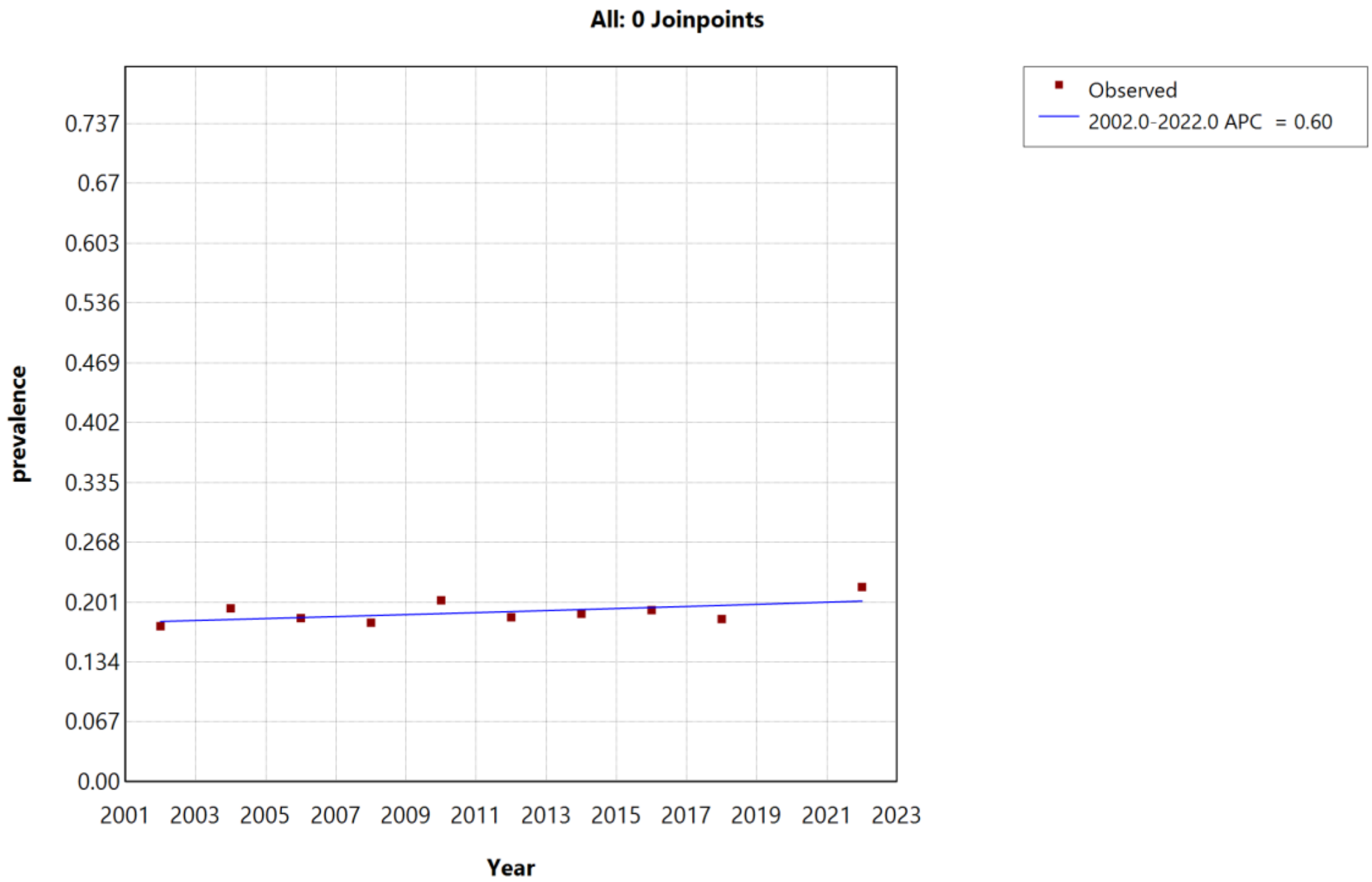

* Indicates that the Annual Percent Change (APC) is significantly different from zero at the alpha = 0.05 level.
Final Selected Model: 0 Joinpoints.

Section 12: Joinpoints in the female subgroup
Note. The observed prevalence values and their 95% confidence intervals are provided in Section 29.

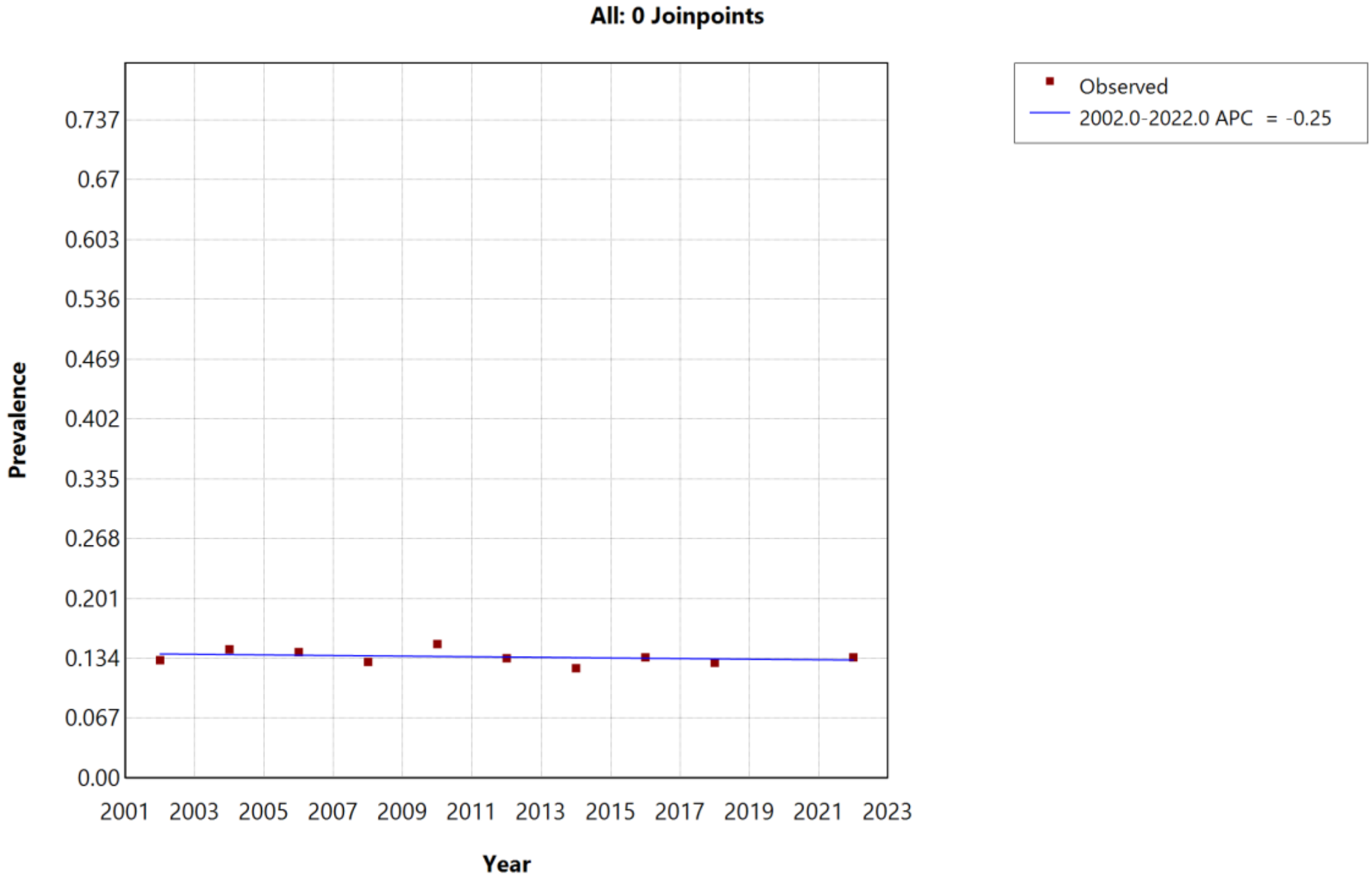

* Indicates that the Annual Percent Change (APC) is significantly different from zero at the alpha = 0.05 level.
Final Selected Model: 0 Joinpoints.

Section 13: Joinpoints in the male subgroup

Note. The observed prevalence values and their 95% confidence intervals are provided in Section 29.

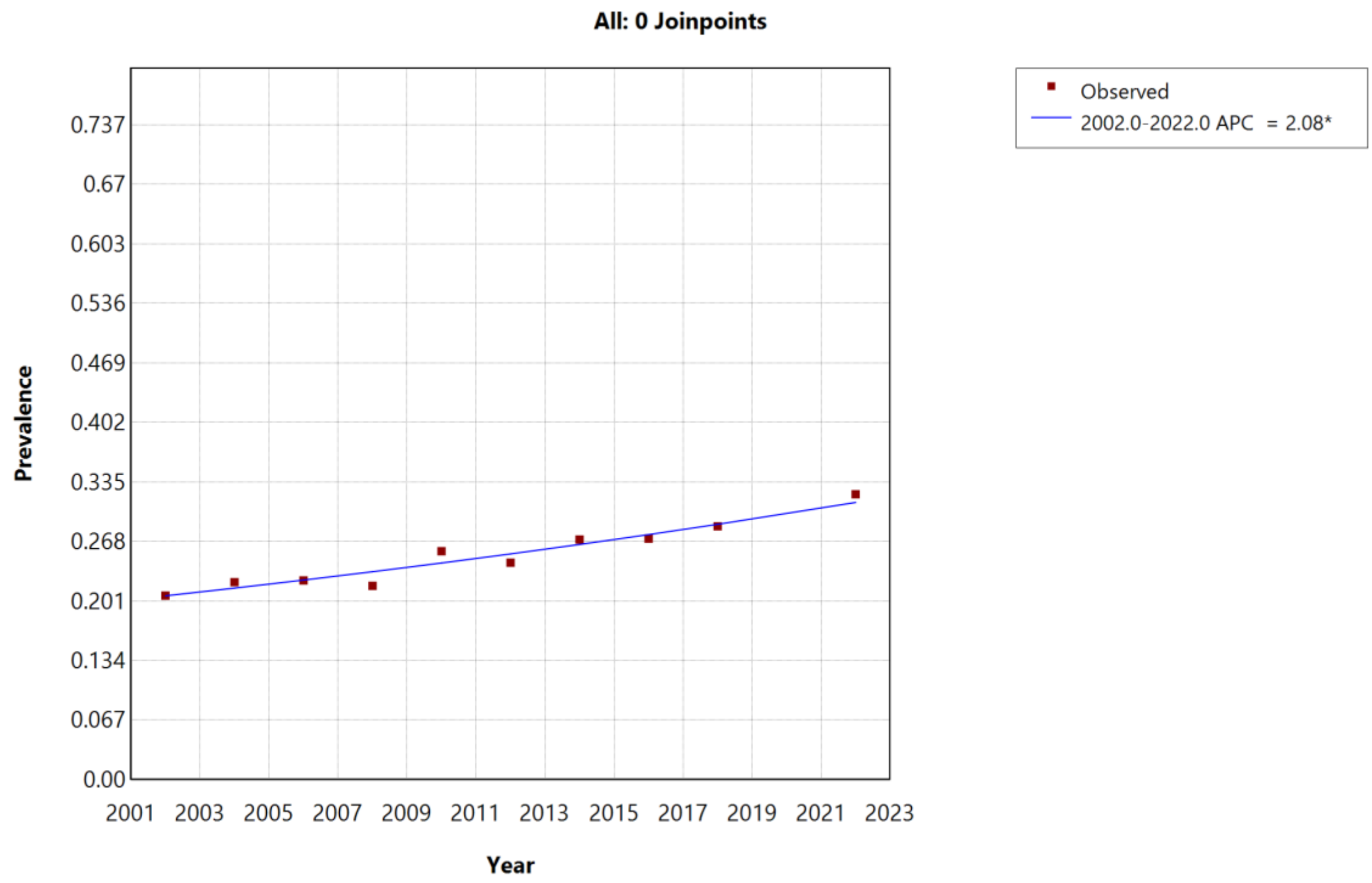

* Indicates that the Annual Percent Change (APC) is significantly different from zero at the alpha = 0.05 level.
Final Selected Model: 0 Joinpoints.

Section 14: Joinpoints in the subgroup of lower education level
Note. The observed prevalence values and their 95% confidence intervals are provided in Section 29.

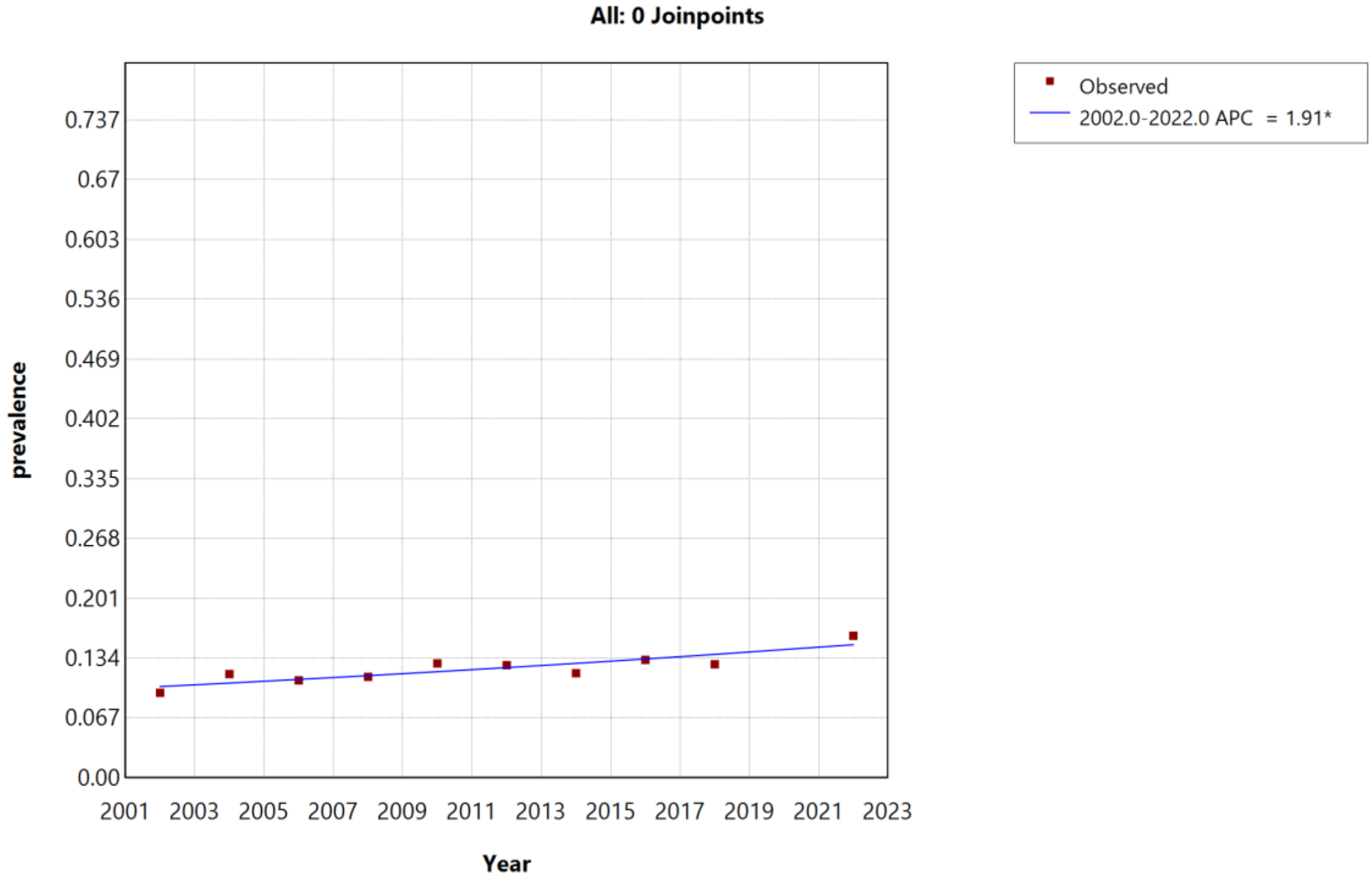

* Indicates that the Annual Percent Change (APC) is significantly different from zero at the alpha = 0.05 level.
Final Selected Model: 0 Joinpoints.

Section 15: Joinpoints in the subgroup of intermediate education level
Note. The observed prevalence values and their 95% confidence intervals are provided in Section 29.

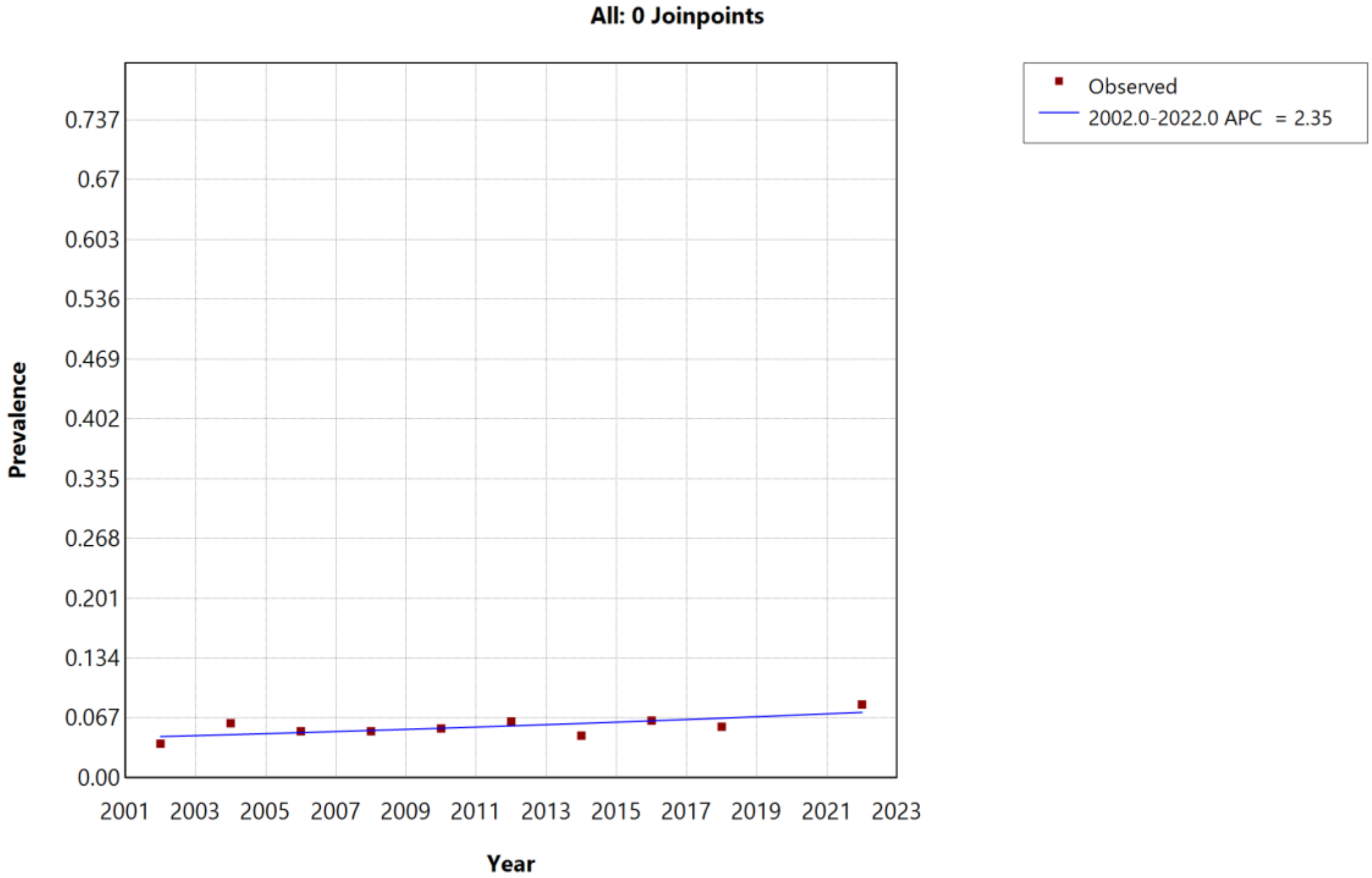

* Indicates that the Annual Percent Change (APC) is significantly different from zero at the alpha = 0.05 level.
Final Selected Model: 0 Joinpoints.

Section 16: Joinpoints in the subgroup of higher education level

Note. The observed prevalence values and their 95% confidence intervals are provided in Section 29.

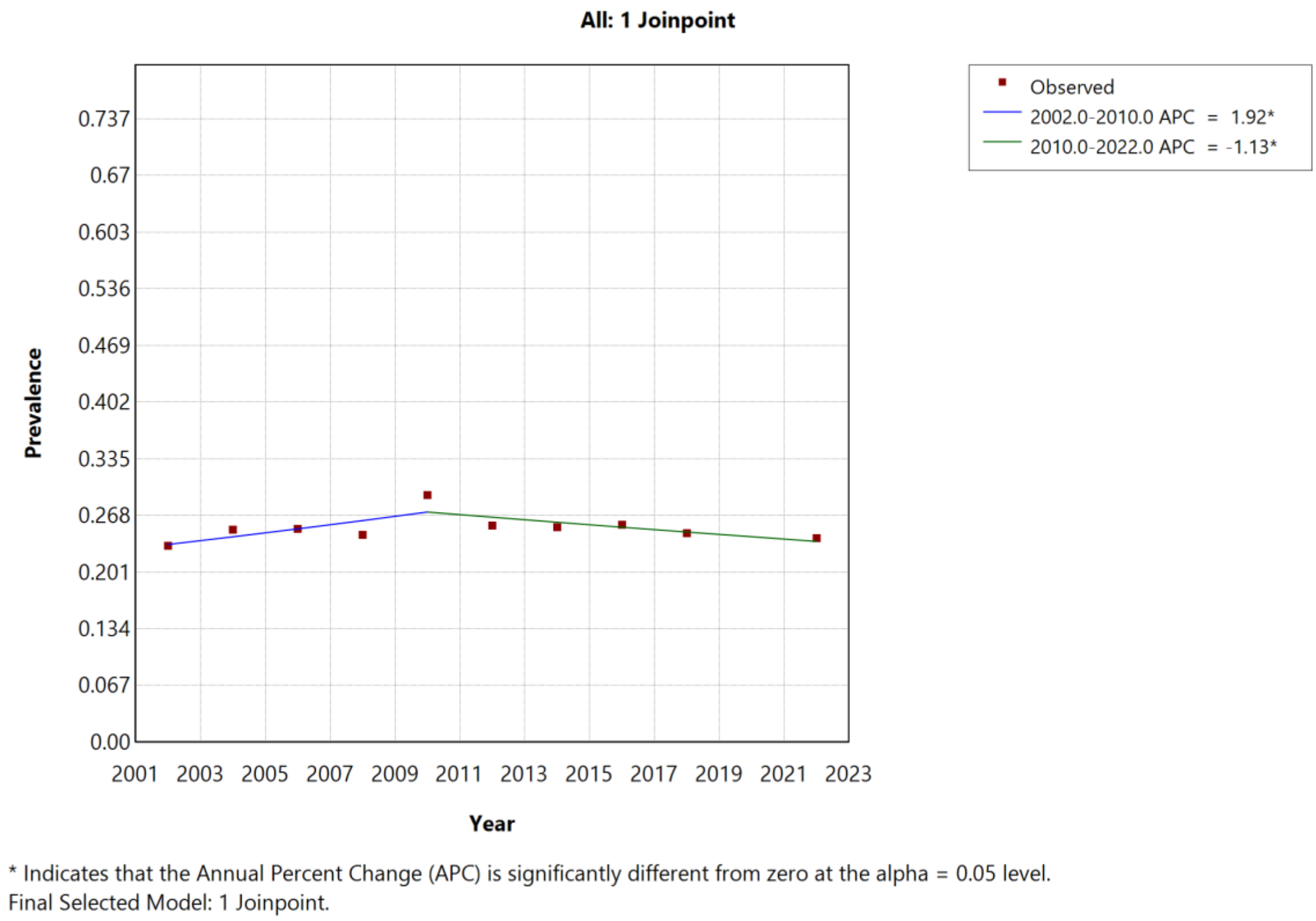

Section 17: Joinpoints in the subgroup of being single

Note. The observed prevalence values and their 95% confidence intervals are provided in Section 29.

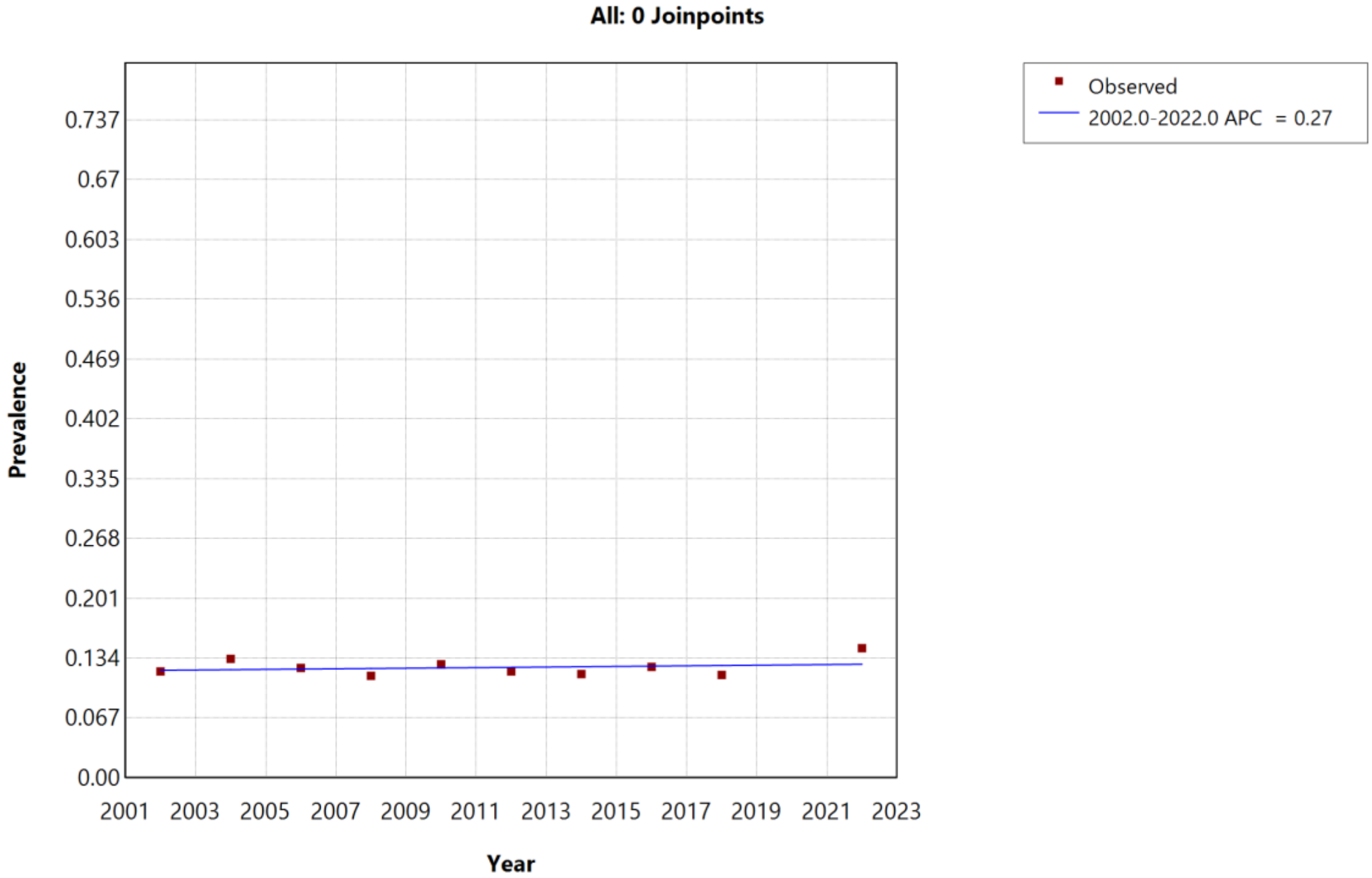

* Indicates that the Annual Percent Change (APC) is significantly different from zero at the alpha = 0.05 level.
Final Selected Model: 0 Joinpoints.

Section 18: Joinpoints in the subgroup of being partnered
Note. The observed prevalence values and their 95% confidence intervals are provided in Section 29.

Section 19: Associations of explanatory factors with frailty

	Coefficient	P	95% CI	
			Lower 95% CI	Upper 95% CI
Age (ref: 50-64)				
65-74	0·016	P<0·001	0·009	0·023
75-84	0·061	P<0·001	0·050	0·073
≥85	0·176	P<0·001	0·154	0·198
Sex : Female	0·015	0·002	0·007	0·024
Education (ref: lower level)				
Intermediate level	-0·055	P<0·001	-0·066	-0·045
Higher level	-0·070	P<0·001	-0·080	-0·059
Marital status: Single	0·044	P<0·001	0·034	0·054
MET	-0·035	P<0·001	-0·036	-0·033
Smoking: Yes	0·026	P<0·001	0·018	0·035
House problems	0·035	P<0·001	0·031	0·040
SDI	-0·840	0·135	-1·943	0·263
Year	0·005	0·006	0·001	0·009

N.B. SDI: Sociodemographic development index; MET: Metabolic equivalent score.

Section 20: Changes of explanatory factors between 2002 and 2022

	2002	2022	Difference
Age (ref: 50-64)			
65-74	26·0%	25·4%	-0·5%
75-84	16·9%	17·0%	0·1%
≥85	4·8%	5·9%	1·2%
Sex: Female	53·6%	52·3%	-1·3%
Education (ref: lower level)			
Intermediate level	28·9%	52·1%	23·2%
Higher level	11·6%	24·5%	12·9%
Marital status: Single	31·4%	34·5%	3·1%
MET	5·37	5·67	0·30
Smoking: Yes	64·4%	58·4%	-6·0%
Housing problems	0·56	0·57	0·01
SDI	0·80	0·86	0·06

Section 21: Contribution of different factors to changes in frailty prevalence in England between 2002 and 2022

	Mean difference between 2002 and 2022	Explained change	Proportion of total change, %
Changes in frailty	/	0·023	100%
Age (ref: 50-64)	/	0·002	8·74%
65-74	0·062	0·000	-0·35%
75-84	0·069	0·000	0·27%
≥85	0·033	0·002	8·83%
Sex : Female	0·020	0·000	-0·88%
Education (ref: lower level)	/	-0·022	-94·90%
Intermediate level	0·188	-0·013	-55·91%
Higher level	0·152	-0·009	-38·98%
Marital status: Single	0·047	0·001	5·97%
MET	0·279	-0·010	-44·80%
Smoking: Yes	-0·062	-0·002	-6·82%
House problems	-0·076	0·000	0·86%
SDI	0·061	-0·051	-222·47%
Year	20	0·101	440·59%
Total explained change	/	0·020	86·30%
Total unexplained change	/	0·003	13·70%

Section 22: Projected prevalence of frailty and frail population in England in 2040

	2022	2040
Age (ref: 50-64)		
65-74	25·45%	26·93%
75-84	16·99%	20·67%
≥85	5·91%	9·47%
Sex : Female	52·30%	52·36%
Education (ref: lower level)		
Intermediate level	52·09%	48·59%
Higher level	24·46%	27·21%
Marital status: Single	34·51%	33·33%
MET	5·67	6·25
Smoking: Yes	58·41%	43·61%
Housing problems	0·57	0·60
SDI	0·86	0·92
Year	2022	2040
Prevalence	17·8%	20·5%
Frail population	3791038	5213689

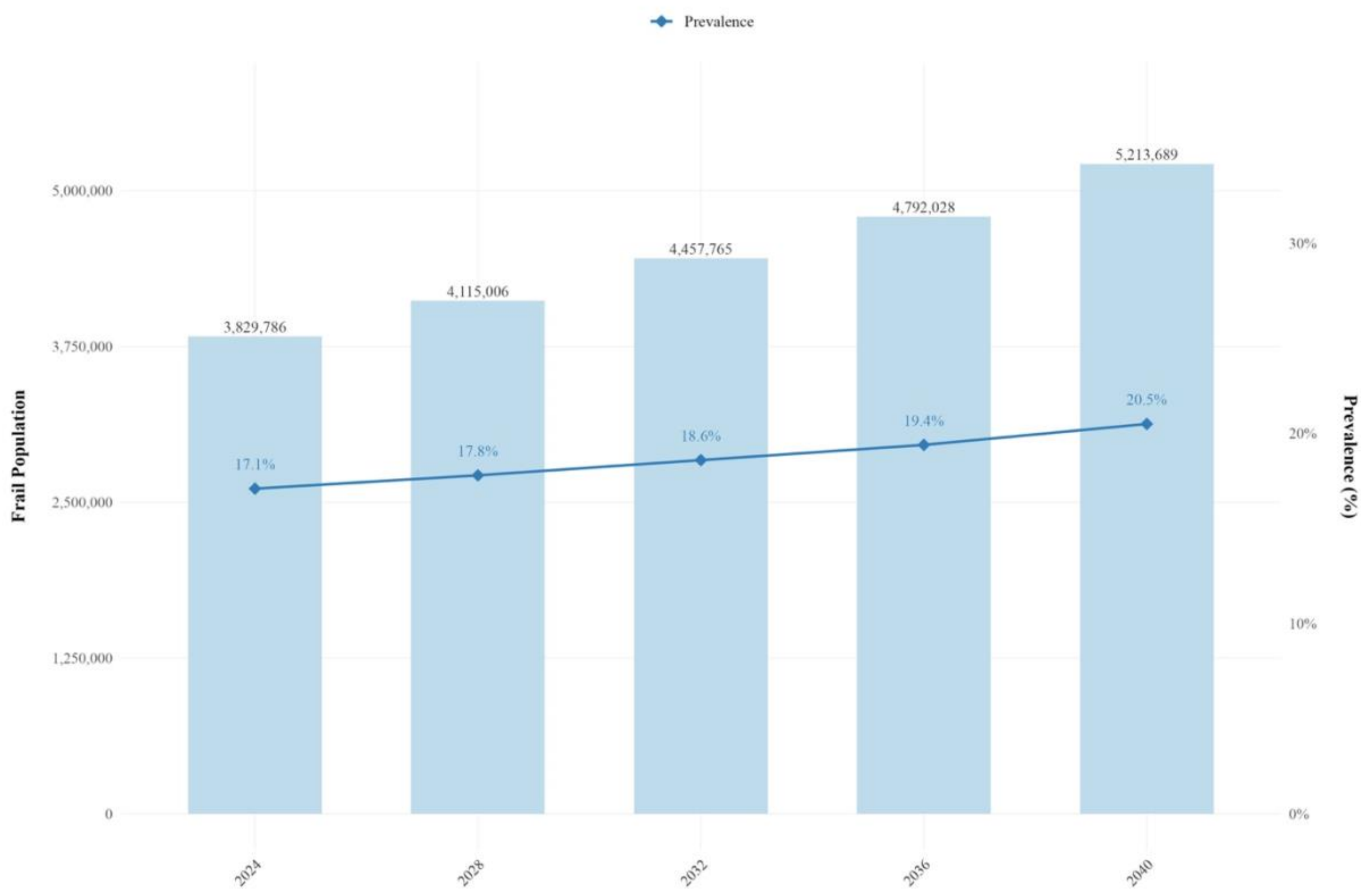

Section 23: Projected prevalence of frailty from 2024 to 2040

Section 24: Associations of explanatory factors with frailty using logistic regression model

	Coefficient	P	95% CI	
			Lower 95% CI	Upper 95% CI
Age (ref: 50-64)				
65-74	0.025	P<0.001	0.018	0.033
75-84	0.048	P<0.001	0.039	0.058
≥85	0.079	P<0.001	0.065	0.093
Sex : Female	0.019	P<0.001	0.011	0.028
Education (ref: lower level)				
Intermediate level	-0.042	P<0.001	-0.051	-0.033
Higher level	-0.081	P<0.001	-0.093	-0.068
Marital status: Single	0.032	P<0.001	0.024	0.040
MET	-0.046	P<0.001	-0.048	-0.045
Smoking: Yes	0.027	P<0.001	0.019	0.035
House problems	0.029	P<0.001	0.026	0.032
SDI	-1.090	0.042	-2.139	-0.041
Year	0.005	0.002	0.002	0.009

Section 25: Associations of explanatory factors with frailty using modified FI

	Coefficient	P	95% CI	
			Lower 95% CI	Upper 95% CI
Age (ref: 50-64)				
65-74	0.005	0.095	-0.001	0.012
75-84	0.033	P<0.001	0.023	0.044
≥85	0.118	P<0.001	0.098	0.139
Sex : Female	-0.006	0.002	-0.097	-0.001
Education (ref: lower level)				
Intermediate level	-0.035	P<0.001	-0.044	-0.027
Higher level	-0.045	P<0.001	-0.054	-0.035
Marital status: Single	0.034	P<0.001	0.025	0.042
MET	-0.026	P<0.001	-0.027	-0.025
Smoking: Yes	0.020	P<0.001	0.013	0.027
House problems	0.029	P<0.001	0.024	0.033
SDI	-0.188	0.711	-1.184	0.807
Year	0.003	0.067	0.000	0.006

Section 26: Contribution of different factors to changes in frailty prevalence in England between 2002 and 2022 using modified FI

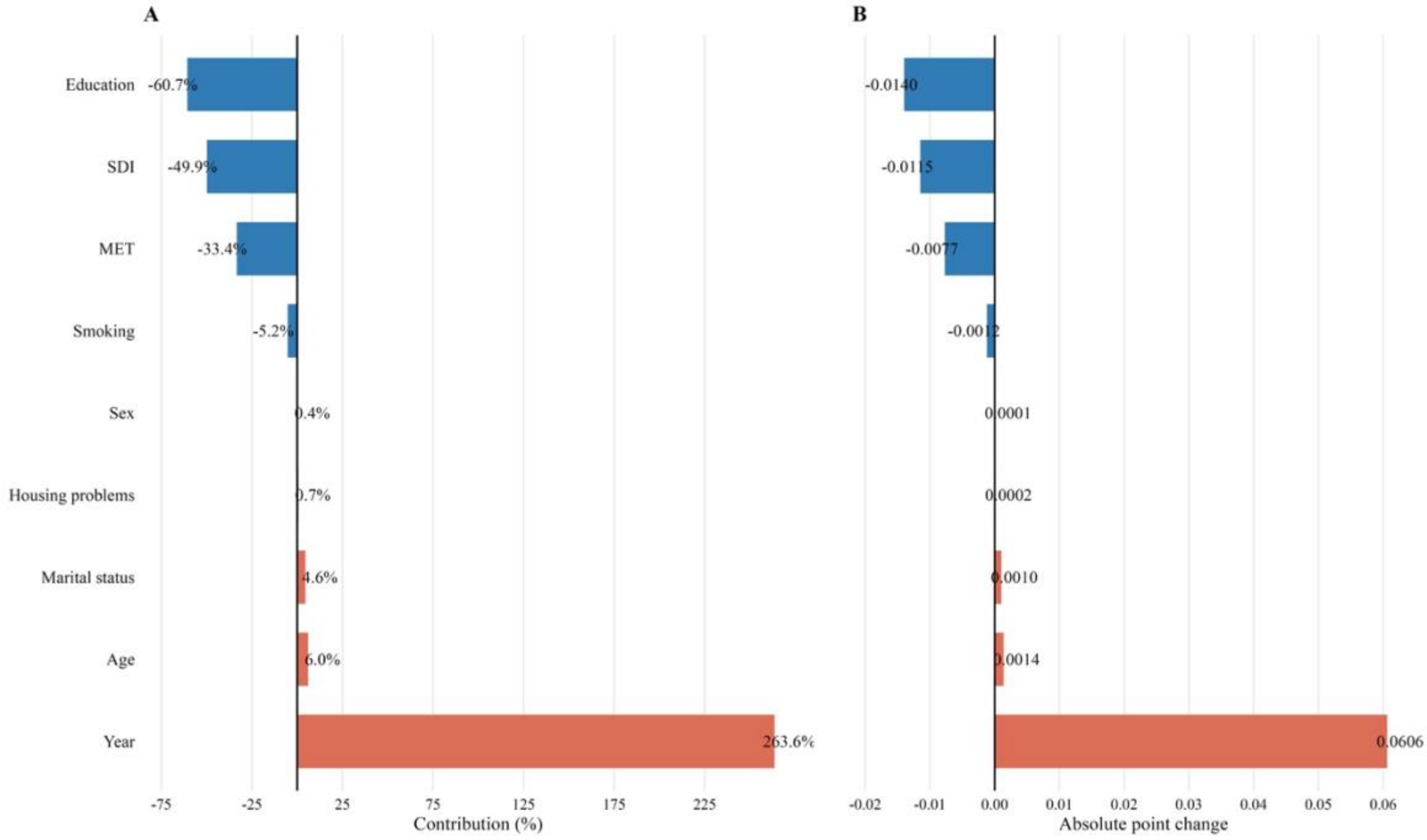

Section 27: Associations of explanatory factors with frailty after excluding wave 10

	Coefficient	P	95% CI	
			Lower 95% CI	Upper 95% CI
Age (ref: 50-64)				
65-74	0.018	P<0.001	0.010	0.026
75-84	0.063	P<0.001	0.050	0.075
≥85	0.180	P<0.001	0.157	0.203
Sex : Female	0.012	0.006	0.003	0.021
Education (ref: lower level)				
Intermediate level	-0.054	P<0.001	-0.065	-0.044
Higher level	-0.069	P<0.001	-0.080	-0.058
Marital status: Single	0.046	P<0.001	0.035	0.056
MET	-0.035	P<0.001	-0.036	-0.033
Smoking: Yes	0.024	P<0.001	0.015	0.033
House problems	0.034	P<0.001	0.030	0.039
SDI	-0.916	0.136	-2.121	0.289
Year	0.005	0.011	0.001	0.009

Section 28: Contribution of different factors to changes in frailty prevalence in England between 2002 and 2018

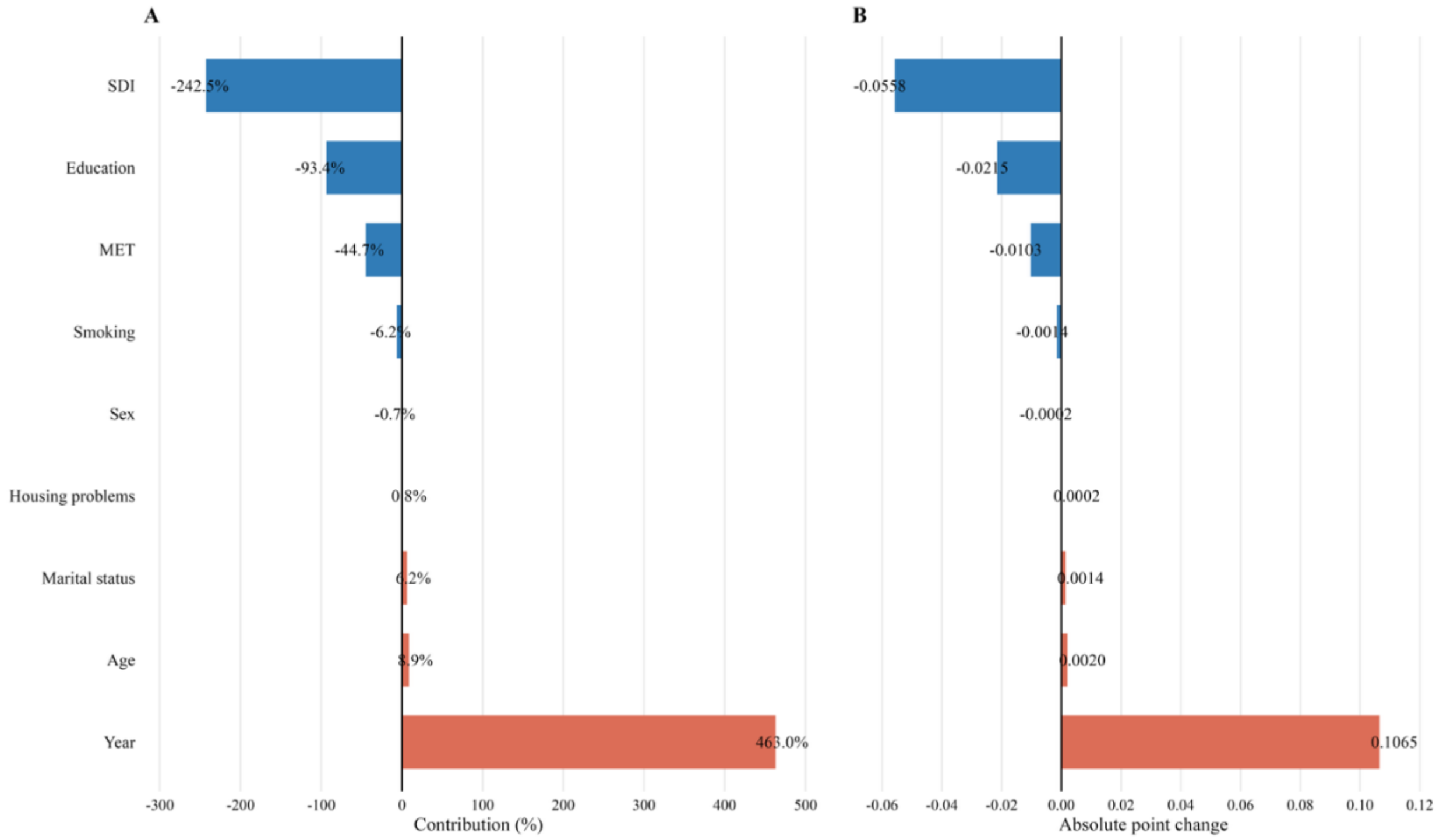

Section 29: Frailty prevalence with 95% CI by year, overall and by subgroup

Subgroup	2002	2004	2006	2008	2010	2012	2014	2016	2018	2022
**Overall**	0.155 (0.148-0.161)	0.171 (0.163-0.179)	0.163 (0.156-0.171)	0.155 (0.148-0.162)	0.178 (0.170-0.186)	0.161 (0.153-0.168)	0.158 (0.150-0.165)	0.165 (0.156-0.174)	0.157 (0.148-0.165)	0.178 (0.169-0.188)
Age 50-64	0.100 (0.092-0.108)	0.106 (0.097-0.116)	0.101 (0.092-0.110)	0.095 (0.087-0.103)	0.106 (0.096-0.115)	0.100 (0.090-0.109)	0.091 (0.080-0.101)	0.100 (0.087-0.113)	0.097 (0.085-0.109)	0.129 (0.115-0.143)
Age 65-74	0.157 (0.144-0.169)	0.158 (0.144-0.172)	0.160 (0.145-0.175)	0.154 (0.141-0.167)	0.165 (0.151-0.178)	0.160 (0.146-0.173)	0.141 (0.129-0.154)	0.154 (0.141-0.168)	0.139 (0.126-0.152)	0.163 (0.148-0.179)
Age 75-84	0.243 (0.224-0.263)	0.266 (0.244-0.287)	0.260 (0.238-0.282)	0.271 (0.248-0.293)	0.285 (0.263-0.307)	0.262 (0.242-0.283)	0.276 (0.255-0.297)	0.248 (0.227-0.268)	0.249 (0.228-0.269)	0.264 (0.241-0.286)
Age ≥85	0.426 (0.379-0.473)	0.442 (0.395-0.490)	0.458 (0.413-0.503)	0.402 (0.358-0.446)	0.461 (0.418-0.504)	0.445 (0.402-0.488)	0.510 (0.466-0.554)	0.486 (0.441-0.532)	0.476 (0.432-0.519)	0.426 (0.380-0.473)
Sex: Male	0.132 (0.123-0.141)	0.144 (0.133-0.155)	0.141 (0.130-0.152)	0.130 (0.120-0.139)	0.150 (0.139-0.161)	0.134 (0.124-0.145)	0.123 (0.113-0.134)	0.135 (0.123-0.147)	0.129 (0.117-0.141)	0.135 (0.122-0.148)
Sex: Female	0.174 (0.165-0.184)	0.194 (0.183-0.205)	0.183 (0.172-0.194)	0.178 (0.167-0.188)	0.203 (0.191-0.214)	0.184 (0.174-0.195)	0.188 (0.177-0.200)	0.192 (0.180-0.204)	0.182 (0.170-0.194)	0.218 (0.204-0.231)
Education: Lower	0.207 (0.197-0.216)	0.222 (0.210-0.233)	0.224 (0.211-0.236)	0.218 (0.206-0.230)	0.257 (0.244-0.270)	0.244 (0.231-0.258)	0.270 (0.255-0.286)	0.271 (0.254-0.288)	0.285 (0.267-0.303)	0.321 (0.298-0.345)
Education: Intermediate	0.095 (0.085-0.105)	0.116 (0.104-0.128)	0.109 (0.098-0.121)	0.113 (0.103-0.124)	0.128 (0.117-0.140)	0.126 (0.116-0.137)	0.117 (0.106-0.128)	0.132 (0.120-0.144)	0.127 (0.116-0.139)	0.159 (0.146-0.172)
Education: Higher	0.038 (0.028-0.049)	0.061 (0.047-0.075)	0.052 (0.039-0.064)	0.052 (0.041-0.062)	0.055 (0.044-0.067)	0.063 (0.051-0.074)	0.047 (0.037-0.058)	0.064 (0.051-0.077)	0.057 (0.046-0.069)	0.082 (0.069-0.096)
Marital status: Partnered	0.119 (0.112-0.126)	0.133 (0.124-0.142)	0.123 (0.115-0.132)	0.114 (0.107-0.122)	0.127 (0.119-0.135)	0.119 (0.111-0.127)	0.116 (0.108-0.125)	0.124 (0.114-0.133)	0.115 (0.106-0.124)	0.145 (0.135-0.156)
Marital status: Single	0.232 (0.218-0.246)	0.251 (0.235-0.267)	0.252 (0.236-0.268)	0.245 (0.230-0.260)	0.292 (0.275-0.308)	0.256 (0.240-0.272)	0.254 (0.237-0.270)	0.257 (0.240-0.275)	0.247 (0.229-0.264)	0.241 (0.223-0.259)

Reference

1. Leopold L, van Valkengoed IGM, Engelhardt H. Education and age trajectories of chronic conditions: Are tests of the cumulative advantage and disadvantage hypothesis biased by underreporting? *Soc Sci Med* 2023; **334**: 116134.
2. Dhalwani NN, O'Donovan G, Zaccardi F, et al. Long terms trends of multimorbidity and association with physical activity in older English population. *Int J Behav Nutr Phys Act* 2016; **13**: 8.
3. Feter N, Dumith SC, Smith EC, et al. Physical activity attenuates the risk for dementia associated with aging in older adults with mild cognitive impairment. Findings from a population-based cohort study. *J Psychiatr Res* 2021; **141**: 1-8.
4. Hamer M, Lavoie KL, Bacon SL. Taking up physical activity in later life and healthy ageing: the English longitudinal study of ageing. *Br J Sports Med* 2014; **48**(3): 239-43.
5. Kim HJ, Fay MP, Feuer EJ, Midthune DN. Permutation tests for joinpoint regression with applications to cancer rates. *Stat Med* 2000; **19**(3): 335-51.
6. Luo Y, Wang Q, Wang Y, et al. Trends in the prevalence and burden of blindness and vision loss among adolescents aged 10-24 years in the Western Pacific Region over 30 years. *BMC Public Health* 2024; **24**(1): 3193.
7. ONS. National population projections: 2022-based. 2025. https://www.ons.gov.uk/peoplepopulationandcommunity/populationandmigration/populationprojections/bulletins/nationalpopulationprojections/2022based (accessed 04.01 2025).
